# Supplementary material for: Modelling the persistence and control of Rift Valley fever virus in a spatially heterogeneous landscape
Source: Nat Commun. 2021 Sep 22;12:5593. doi: 10.1038/s41467-021-25833-8 (PMC8458460; doi:10.1038/s41467-021-25833-8)
Supplement: Supplementary file 1 — Supplementary Information [file 41467_2021_25833_MOESM1_ESM.pdf]

Modelling the persistence and control of Rift Valley fever virus in a spatially heterogeneous landscape

Supplementary information

Warren S. D. Tennant[1,2,*] Eric Cardinale[3,4] Catherine Cêtre-Sossah[3,4]
Youssouf Moutroifi[5] Gilles Le Godais[6] Davide Colombi[7]
Simon E. F. Spencer[1,8] Mike J. Tildesley[1,2,9] Matt J. Keeling[1,2,9]
Onzade Charafouddine[5] Vittoria Colizza[10] W. John Edmunds[11]
Raphaëlle Métras[10,11]

[1]The Zeeman Institute: SBIDER, University of Warwick, Coventry CV4 7AL, United Kingdom

[2]Mathematics Institute, University of Warwick, Coventry CV4 7AL, United Kingdom

[3]Centre de Coopération Internationale en Recherche Agronomique pour le Développement, UMR Animal, Santé, Territoires, Risques, et Écosystèmes, F-97490 Sainte Clotilde, La Réunion, France

[4]Animal, Santé, Territoires, Risques, et Écosystèmes, Université de Montpellier, Centre de Coopération Internationale en Recherche Agronomique pour le Développement, INRAE, Montpellier, France

[5]Vice-Présidence en charge de l'Agriculture, l'Elevage, la Pêche, l'Industrie, l'Energie et l'Artisanat, B.P. 41 Mdé, Moroni, Union des Comores

[6]Direction de l'Alimentation, de l'Agriculture et de la Forêt de Mayotte, Service de l'Alimentation, 97600 Mamoudzou, France

[7]Aizoon Technology Consulting, Str. del Lionetto 6, Torino, Italy

[8]Department of Statistics, University of Warwick, Coventry CV4, 7AL, United Kingdom

[9]School of Life Sciences, University of Warwick, Coventry CV4 7AL, United Kingdom

[10]INSERM, Sorbonne Université, Institut Pierre Louis d'Épidémiologie et de Santé Publique (Unité Mixte de Recherche en Santé 1136), 75012 Paris, France

[11]Centre for the Mathematical Modelling of Infectious Diseases, Department of Infectious Disease Epidemiology, London School of Hygiene and Tropical Medicine, London WC1E 7HT, United Kingdom

[*]Corresponding author: Warren.Tennant@warwick.ac.uk

Supplementary Tables

Supplementary Table 1. **Model selection.** Five models were fitted to the age-stratified sero-surveys from July 2004 to June 2015. The deviance information criterion (DIC) is presented for each model. The model with the lowest DIC was determined to be the best fitted model (indicated by *). The table shows the model identified,underlying assumptions on viral transmission, and whether the seasonal and constant transmission components were the same or different for each island.

Model number	Transmission	Seasonal component for each island (α)	Constant component for each island (β)	DIC
1	Constant	-	Different	1,580
2a	Linear	Different	Same	1,433
2b	Linear	Same	Different	1,578
3a	Exponential	Different	Same	1,386
3b*	Exponential	Same	Different	1,189

Supplementary Table 2. **Summary of estimated parameters for the exponential model (Model 3b).** Parameters of the metapopulation model were estimated by fitting the model to serological surveys conducted on each island through the 2004–2015 period. Shown in the table are the median and 95% credible intervals (CrI) of 10,000 posterior samples for each estimated parameter for the best fitted model (Model 3b).

Parameter	Description	Median estimate [95% CrI]
$48m_{12}$	Annual movement from Grande Comore to Mohéli	331.0 [293.6, 367.2]
$48m_{13}$	Annual movement from Grande Comore to Anjouan	84.6 [43.0, 126.2]
$48m_{21}$	Annual movement from Mohéli to Grande Comore	657.3 [588.0, 726.4]
$48m_{23}$	Annual movement from Mohéli to Anjouan	62.4 [19.0, 106.5]
$48m_{31}$	Annual movement from Anjouan to Grande Comore	628.8 [565.1, 689.2]
$48m_{32}$	Annual movement from Anjouan to Mohéli	408.0 [364.8, 448.6]
$48m_{34}$	Annual movement from Anjouan to Mayotte	1875.4 [1706.9, 2057.5]
ϵ_1	Proportion immune at time $t = 0$ in Grande Comore	0.39 [0.37, 0.41]
ϵ_2	Proportion immune at time $t = 0$ in Mohéli	0.45 [0.40, 0.50]
ϵ_3	Proportion immune at time $t = 0$ in Anjouan	0.04 [0.02, 0.07]
ϵ_4	Proportion immune at time $t = 0$ in Mayotte	0.14 [0.11, 0.17]
a	Seasonal transmission component scalar for all islands	8.5 [8.1, 8.9]
b_1	Transmission constant for Grande Comore	-0.85 [-0.91, -0.80]
b_2	Transmission constant for Mohéli	-0.89 [-0.96, -0.82]
b_3	Transmission constant for Anjouan	-1.18 [-1.25, -1.12]
b_4	Transmission constant for Mayotte	-1.14 [-1.21, -1.08]
$t^{(\text{imp})}_{\text{start}}$	Start of imports into Grande Comore (epi. weeks since July 2004)	122 [117, 132]
$t^{(\text{imp})}_{\text{duration}}$	Duration of imports into Grande Comore	23.6 [10.7, 36.2]
$48I^{(\text{imp})}$	Annual infectious imports into Grande Comore	199.2 [63.0, 341.0]

Supplementary Table 3. **Summary statistics of the seasonal reproduction number.** The seasonal reproduction number, R_{st}, for RVF based on our fitted model was calculated for each island. The table shows the median and 95% credible interval for the geometric mean seasonal reproduction number over the study period, minimum and maximum annual R_{st} and the number of (calendar) weeks per year for which $R_{st} > 1$ on each island. Estimates were calculated from 1,000 simulations of the best fitted model (Model 3b).

Island	Mean seasonal reproduction number R_{st}	Minimum annual R_{st}	Maximum annual R_{st}	Number of calendar weeks $R_{st} > 1$
Grande Comore	1.69 [1.66, 1.72]	0.87 [0.46, 1.12]	3.99 [3.17, 4.72]	39.1 [38.9, 39.8]
Mohéli	1.59 [1.54, 1.64]	0.87 [0.44, 1.38]	3.40 [2.33, 5.54]	40.4 [38.8, 41.4]
Anjouan	1.17 [1.15, 1.19]	0.48 [0.33, 0.77]	2.95 [2.15, 3.83]	28.1 [27.5, 28.5]
Mayotte	1.07 [1.04, 1.10]	0.48 [0.34, 0.70]	2.77 [2.36, 3.14]	25.9 [25.2, 26.3]

Supplementary Table 4. **Prior distributions for estimated parameters.** The parameters estimated by fitting the metapopulation model to serological surveys from 2004–2015. Priors were chosen based on consultation with the Comorian veterinary services and current and historical understanding of RVF in the Comoros.

Parameter	Description	Prior
$48m_{12}$	Annual movement from Grande Comore to Mohéli	$\text{Normal}\left(350, \frac{350}{10}\right)$
$48m_{13}$	Annual movement from Grande Comore to Anjouan	$\text{Normal}(50, 50)$
$48m_{21}$	Annual movement from Mohéli to Grande Comore	$\text{Normal}\left(676, \frac{676}{10}\right)$
$48m_{23}$	Annual movement from Mohéli to Anjouan	$\text{Normal}(50, 50)$
$48m_{31}$	Annual movement from Anjouan to Grande Comore	$\text{Normal}\left(625, \frac{625}{10}\right)$
$48m_{32}$	Annual movement from Anjouan to Mohéli	$\text{Normal}\left(409, \frac{409}{10}\right)$
$48m_{34}$	Annual movement from Anjouan to Mayotte	$\text{Normal}\left(1500, \frac{1500}{10}\right)$
ϵ_1	Proportion immune at time $t = 0$ in Grande Comore	$\text{Beta}(20, 30)$
ϵ_2	Proportion immune at time $t = 0$ in Mohéli	$\text{Beta}(20, 30)$
ϵ_3	Proportion immune at time $t = 0$ in Anjouan	$\text{Beta}(5, 45)$
ϵ_4	Proportion immune at time $t = 0$ in Mayotte	$\text{Beta}(5, 45)$
$b_i^{(\text{const})}$	Transmission constant for island i (constant)	$\text{Normal}(1, 2)$
$a_i^{(\text{linear})}$	NDVI scalar for island i (linear)	$\text{Normal}(3, 2)$
$b_i^{(\text{linear})}$	Transmission constant for island i (linear)	$\text{Normal}(1, 2)$
$a_i^{(\text{exp})}$	NDVI scalar for island i (exponential)	$\text{Normal}(3, 2)$
$b_i^{(\text{exp})}$	Transmission constant for island i (exponential)	$\text{Normal}(-2, 2)$
$t_{\text{start}}^{(\text{imp})}$	Start of imports into Grande Comore	$\text{Normal}(128, 24)$
$t_{\text{duration}}^{(\text{imp})}$	Duration of imports into Grande Comore	$\text{Normal}(24, 12)$
$48I^{(\text{imp})}$	Annual infectious imports into Grande Comore	$\text{Normal}(150, 150)$

Supplementary Figures

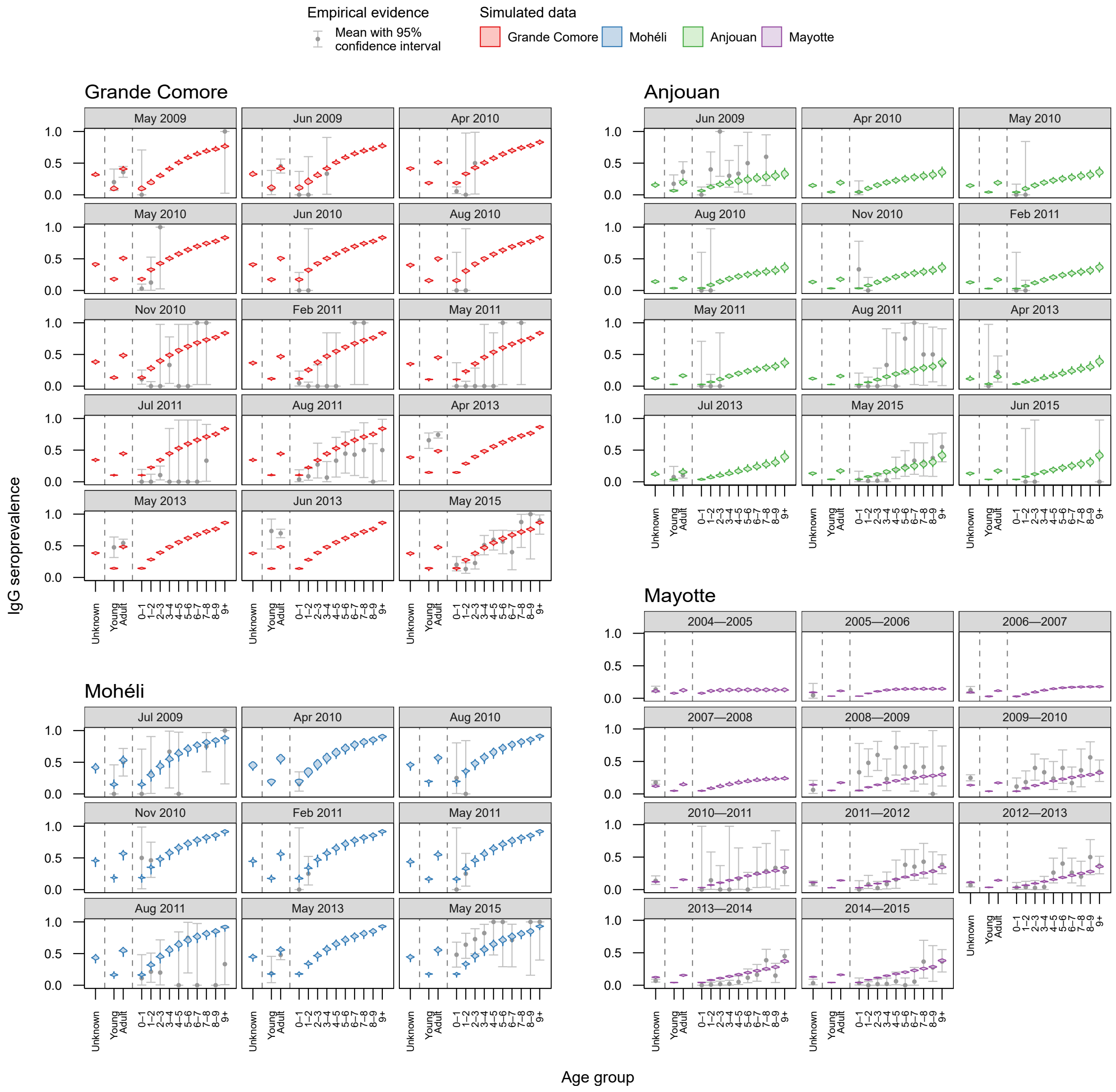

Supplementary Figure 1. **Model fit of the constant transmission model (Model 1) to each sero-survey conducted between July 2004 and June 2015.** The constant transmission model with the different constant transmission β for each island fitted to the data worst out of all five models tested (DIC $= 1,580$). Shown is the fitted simulated seroprevalence for each aggregated sero-survey conducted throughout the study period (coloured violins). Seroprevalence of Grande Comore (red), Mohéli (blue) and Anjouan (green) were aggregated by month, and seroprevalence for Mayotte (purple) was aggregated by year. Simulated seroprevalence was generated through 1,000 realisations of the metapopulation model. Also shown is the mean observed age-stratified IgG seroprevalence (grey points) with 95% confidence interval (vertical error bars). The presented summary metrics were calculated from the $n = 8423$ biologically independent sera-samples which were used to infer the parameters of the metapopulation model. In particular, there were $n = 2191$ samples for Grande Comore, $n = 475$ samples for Mohéli, $n = 857$ samples for Anjouan and $n = 4900$ samples for Mayotte.

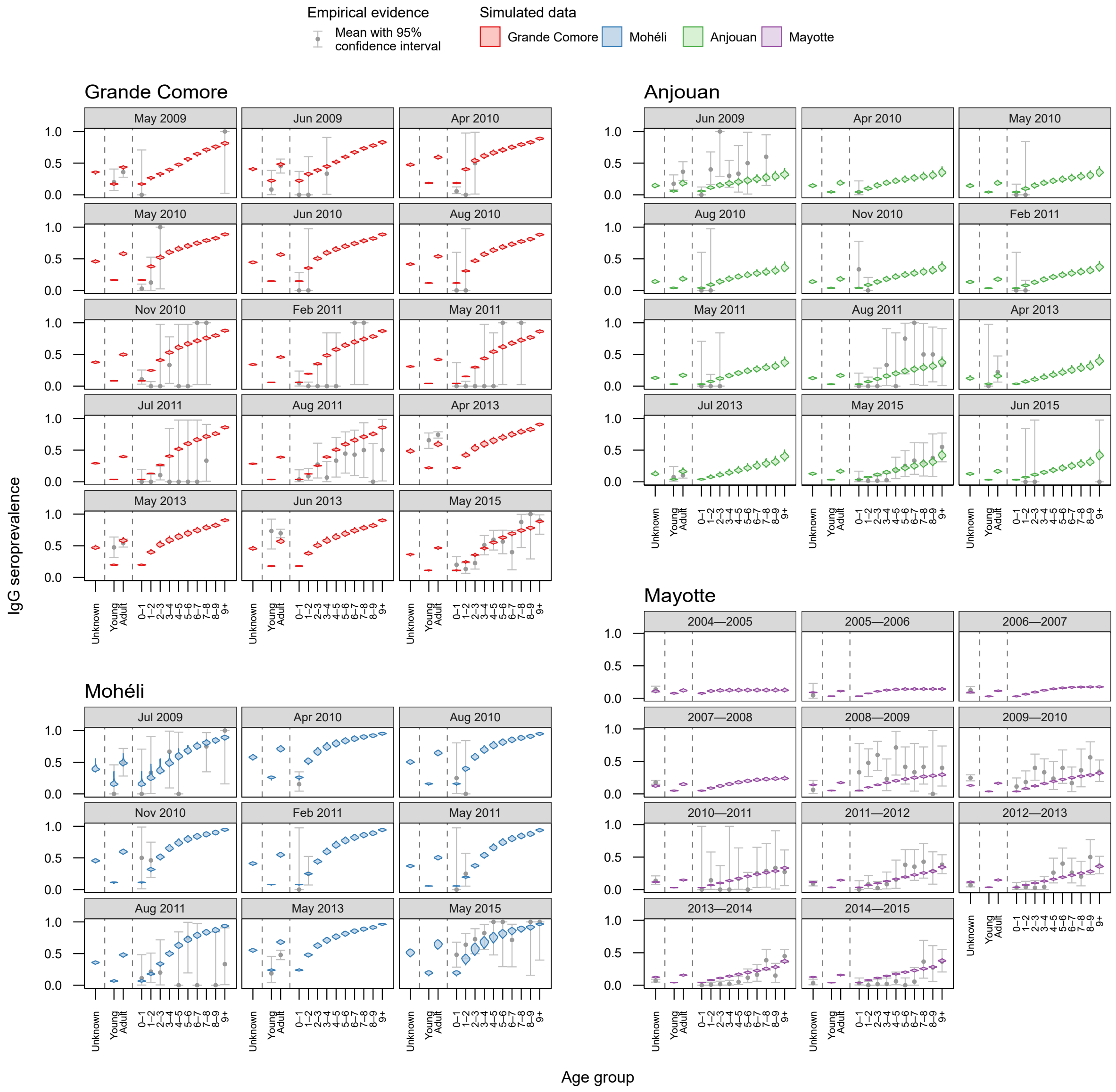

Supplementary Figure 2. **Model fit of the linear model (Model 2a) to each sero-survey conducted between July 2004 and June 2015.** The linear transmission model with the different seasonal components α and the same baseline transmission β for each island fitted to the data third best out of all five tested models (DIC $= 1,433$). Shown is the fitted simulated seroprevalence for each aggregated sero-survey conducted throughout the study period (coloured violins). Seroprevalence of Grande Comore (red), Mohéli (blue) and Anjouan (green) were aggregated by month, and seroprevalence for Mayotte (purple) was aggregated by year. Simulated seroprevalence was generated through 1,000 realisations of the metapopulation model. Also shown is the mean observed age-stratified IgG seroprevalence (grey points) with 95% confidence interval (vertical error bars). The presented summary metrics were calculated from the $n = 8423$ biologically independent sera-samples which were used to infer the parameters of the metapopulation model. In particular, there were $n = 2191$ samples for Grande Comore, $n = 475$ samples for Mohéli, $n = 857$ samples for Anjouan and $n = 4900$ samples for Mayotte.

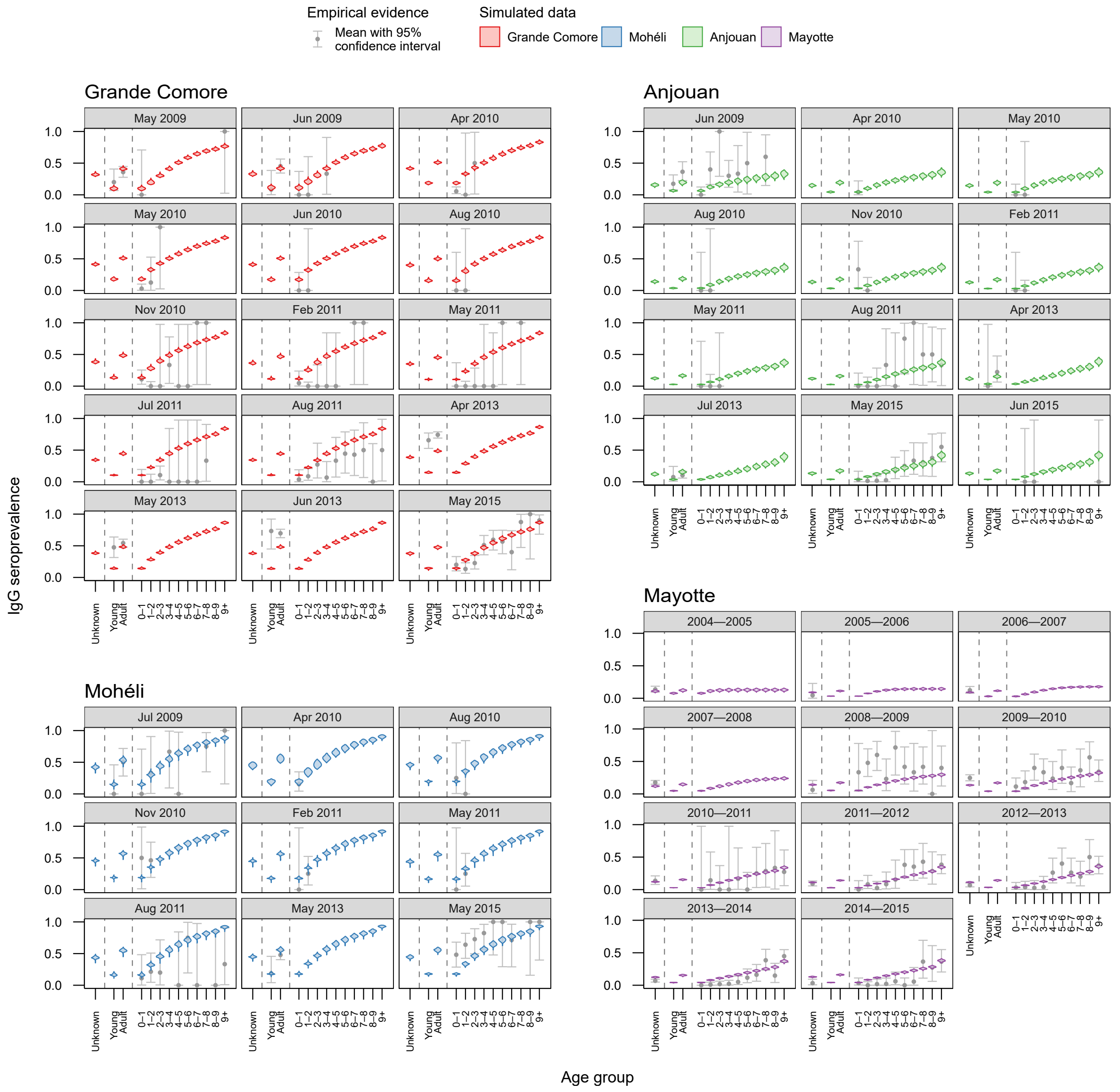

Supplementary Figure 3. **Model fit of the linear model (Model 2b) to each sero-survey conducted between July 2004 and June 2015.** The linear transmission model with the same seasonal component α and different baseline transmission β for each island fitted to the data almost as poorly as the constant transmission model (DIC $= 1,578$). Shown is the fitted simulated seroprevalence for each aggregated sero-survey conducted throughout the study period (coloured violins). Seroprevalence of Grande Comore (red), Mohéli (blue) and Anjouan (green) were aggregated by month, and seroprevalence for Mayotte (purple) was aggregated by year. Simulated seroprevalence was generated through 1,000 realisations of the metapopulation model. Also shown is the mean observed age-stratified IgG seroprevalence (grey points) with 95% confidence interval (vertical error bars). The presented summary metrics were calculated from the $n = 8423$ biologically independent sera-samples which were used to infer the parameters of the metapopulation model. In particular, there were $n = 2191$ samples for Grande Comore, $n = 475$ samples for Mohéli, $n = 857$ samples for Anjouan and $n = 4900$ samples for Mayotte.

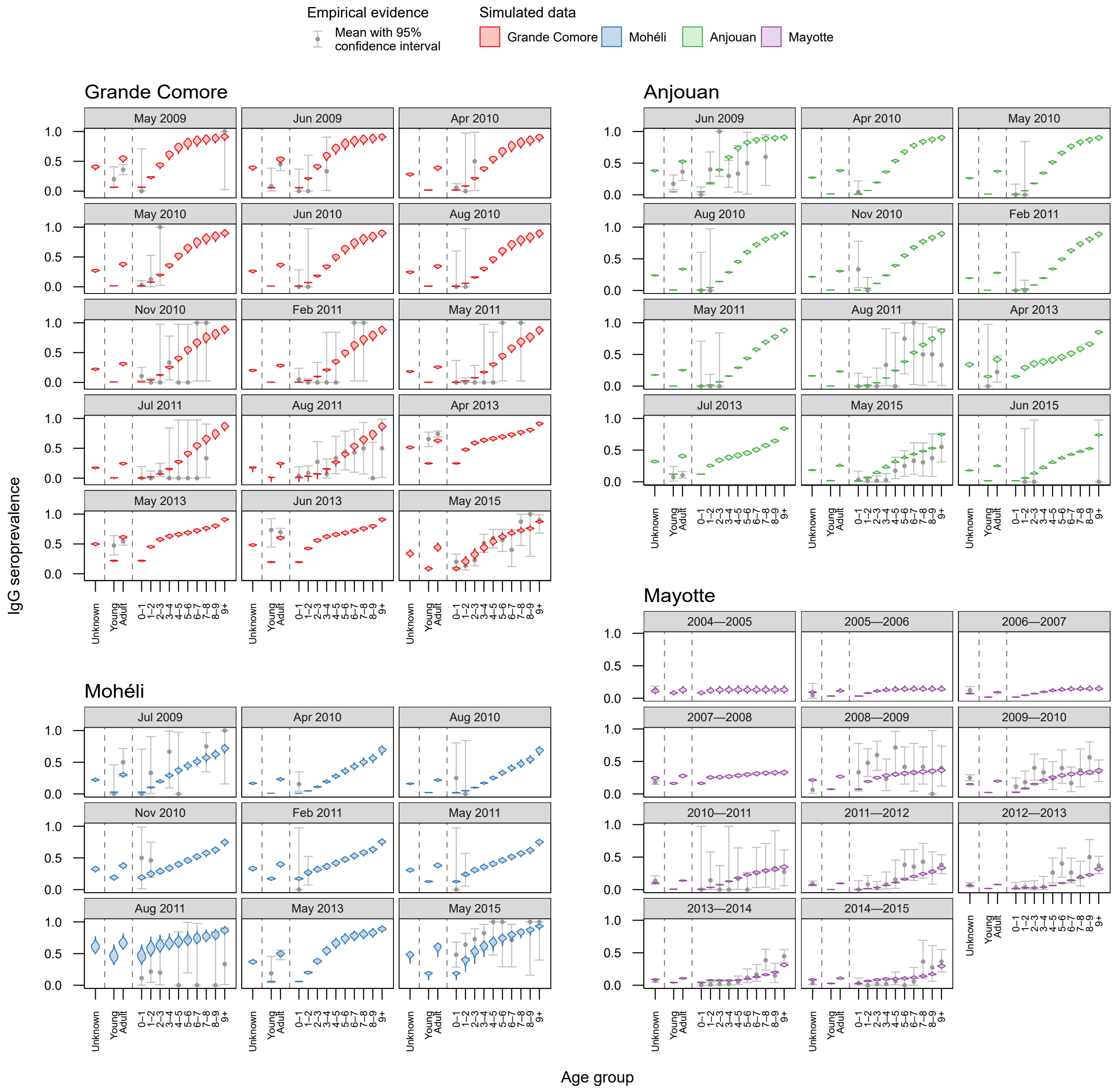

Supplementary Figure 4. **Model fit of the exponential model (Model 3a) to each sero-survey conducted between July 2004 and June 2015.** The exponential transmission model with the same seasonal component α and different baseline transmission β for each island fitted to the data second best out of all five models tested (DIC $= 1,386$). Shown is the fitted simulated seroprevalence for each aggregated sero-survey conducted throughout the study period (coloured violins). Seroprevalence of Grande Comore (red), Mohéli (blue) and Anjouan (green) were aggregated by month, and seroprevalence for Mayotte (purple) was aggregated by year. Simulated seroprevalence was generated through 1,000 realisations of the metapopulation model. Also shown is the mean observed age-stratified IgG seroprevalence (grey points) with 95% confidence interval (vertical error bars). The presented summary metrics were calculated from the $n = 8423$ biologically independent sera-samples which were used to infer the parameters of the metapopulation model. In particular, there were $n = 2191$ samples for Grande Comore, $n = 475$ samples for Mohéli, $n = 857$ samples for Anjouan and $n = 4900$ samples for Mayotte.

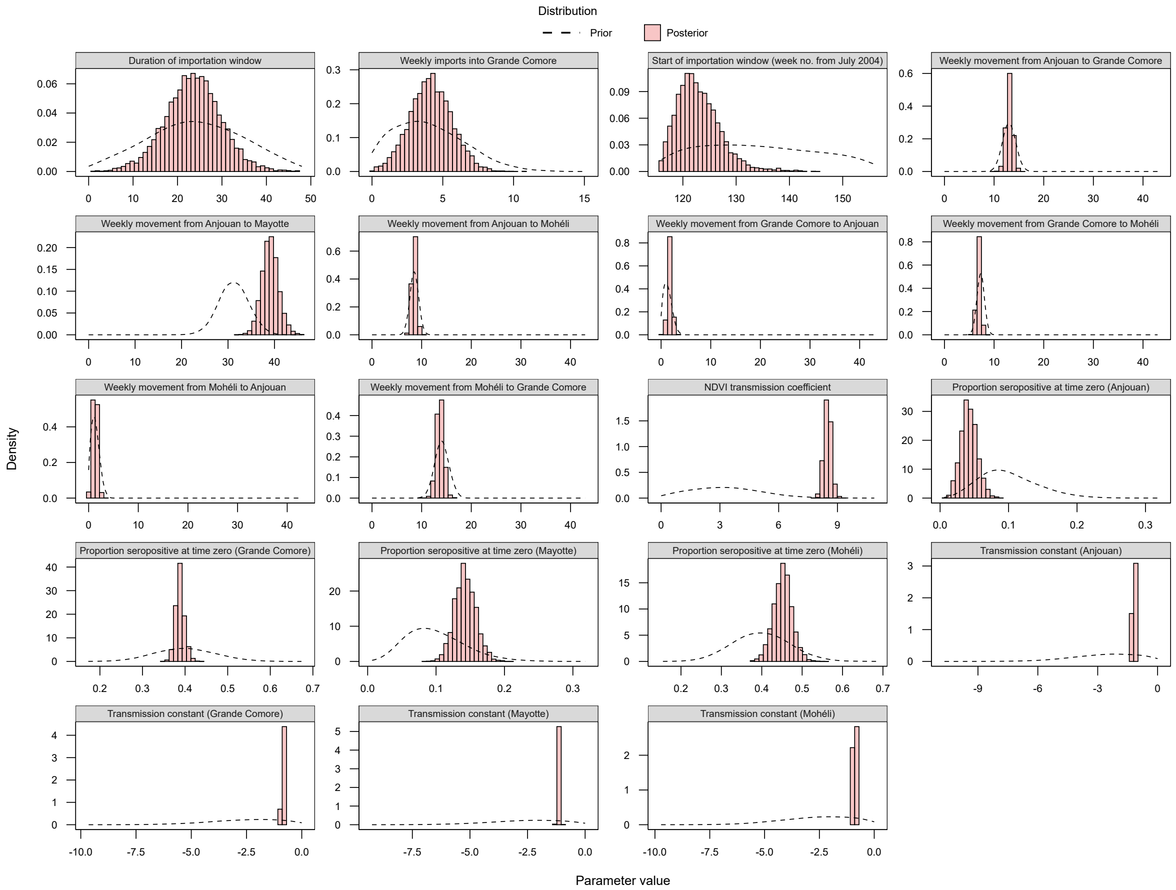

Supplementary Figure 5. **Posterior of estimated parameters in exponential model (Model 3b).** The exponential transmission model with the same seasonal component a and different baseline transmission b for each island fitted to the data best out of all five models tested. Shown are the prior (lines) and posterior (bars) distributions of each parameter. Prior distributions were informed by historical and current understanding of RVF epidemiology and livestock demography in the Comoros. Posterior distributions were generated from 10,000 samples from the fitted model.

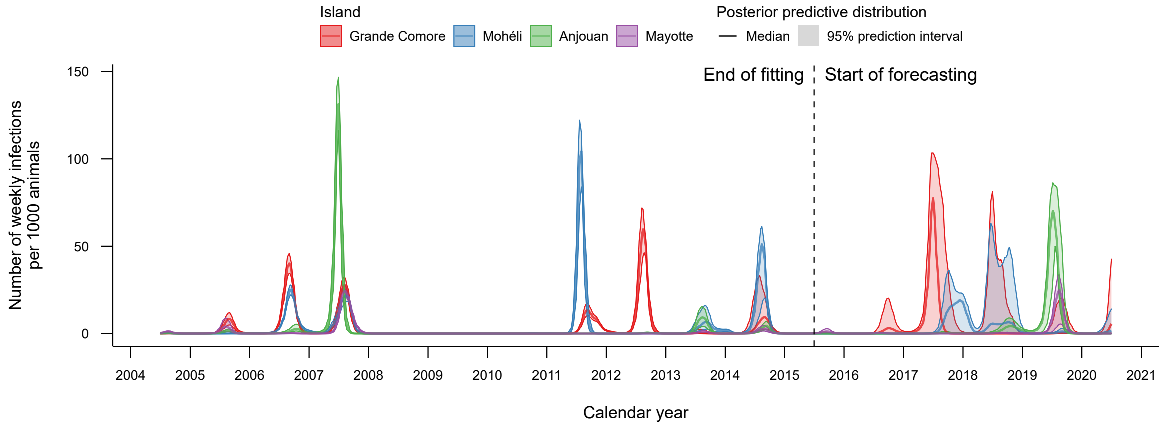

Supplementary Figure 6. **Weekly infections in the Comoros archipelago from 2004 to 2020.** The total number of weekly livestock infections per 1000 animals on each island from July 2004 to July 2020 as predicted by the exponential model (Model 3b). The estimated total livestock population on Grande Comore (red), Mohéli (blue), Anjouan (green) and Mayotte (purple) were 224,353; 31,872; 93,616 and 20,052 respectively. The median and 95% prediction interval of infections was generated through 1,000 realisations of the metapopulation model.

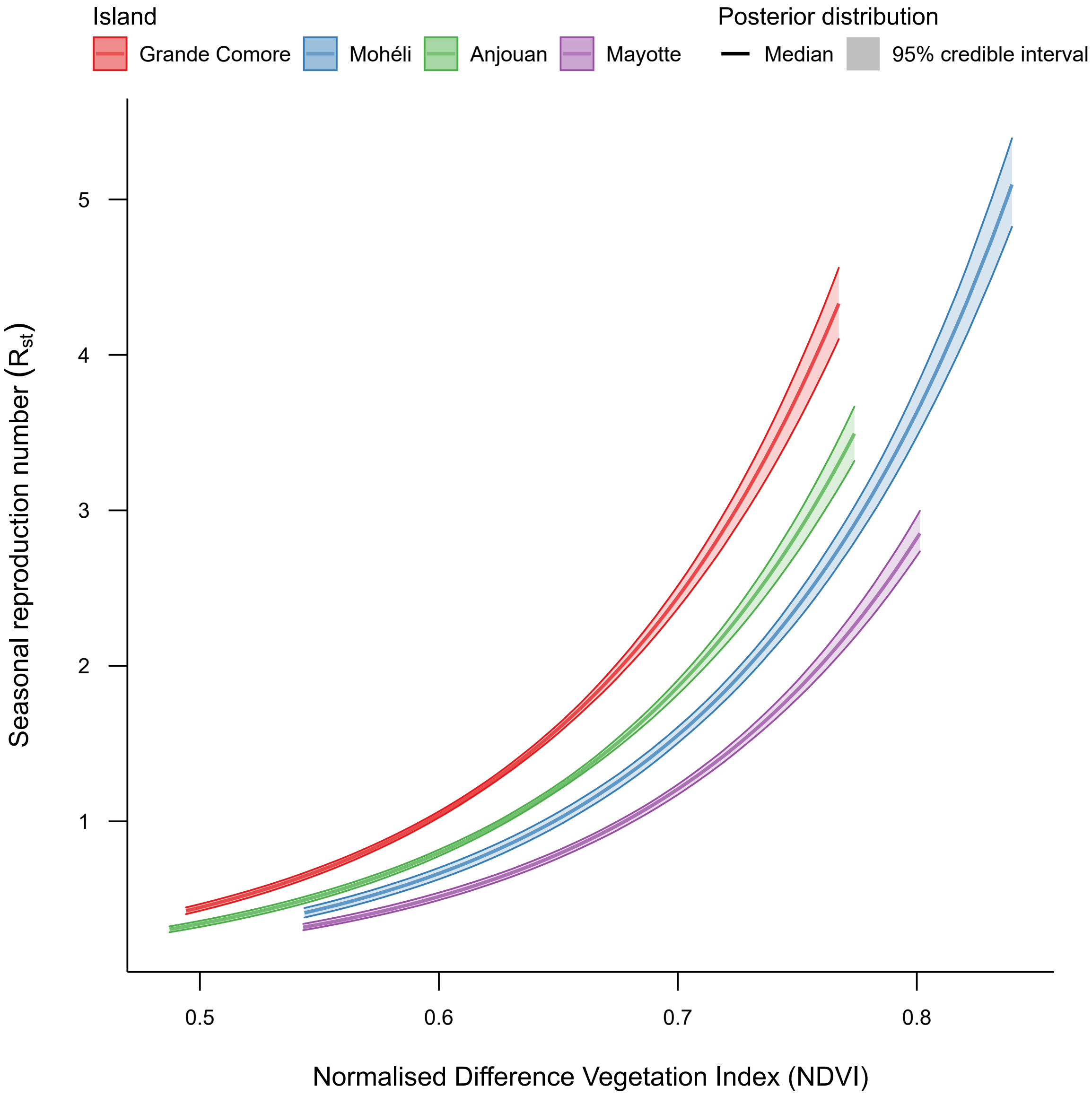

Supplementary Figure 7. **Relationship between NDVI and the seasonal reproduction number in the best fitted model (Model 3b).** In the best fitted model, within-island transmission was modelled as an exponential relationship to Normalized Difference Vegetation Index (NDVI). As a consequence, higher NDVI values resulted in higher seasonal reproduction numbers for the disease on Grande Comore (red), Mohéli (blue), Anjouan (green) and Mayotte (purple). The median and 95% credible interval of the seasonal reproduction number was generated through 1,000 realisations of the metapopulation model.

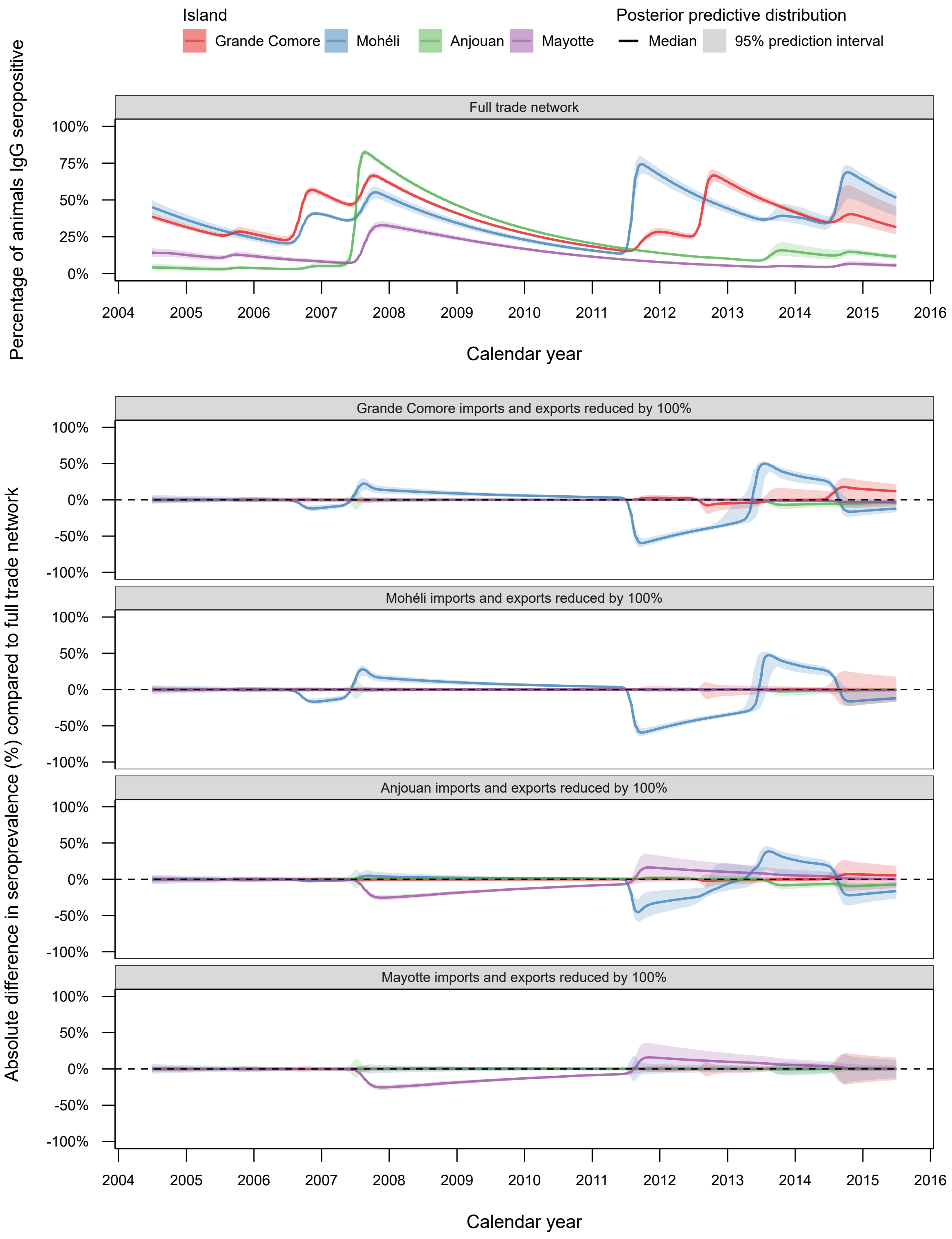

Supplementary Figure 8. **Effect of reducing imports and exports of each island on seroprevalence from 2004–2015.** The best fitted model (Model 3b) was simulated with different levels of import and export restrictions placed on each island. Shown is the absolute difference in the percentage of animals IgG seropositive for RVF from 2004–2015 under each movement restriction scenario on Grande Comore (red), Mohéli (blue), Anjouan (green) and Mayotte (purple) compared with the full trade network model. Distributions were generated from 1,000 realisations of each scenario.

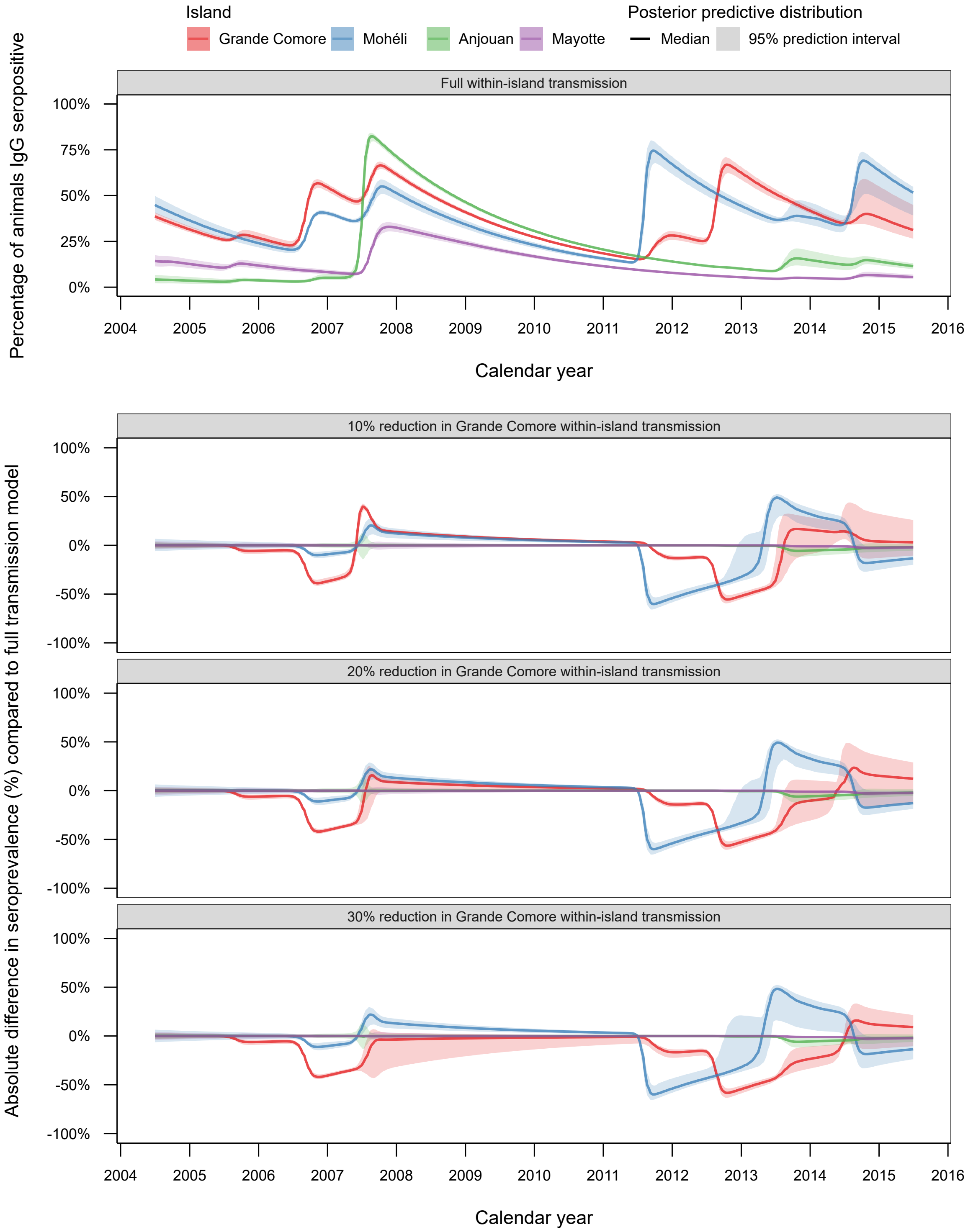

Supplementary Figure 9. **Effect of reducing within-island transmission of Grande Comore on seroprevalence from 2004–2015.** The best fitted model (Model 3b) was simulated with different reductions in the transmission rate on Grande Comore: 10%, 20% and 30%. Shown is the absolute difference in the percentage of animals IgG seropositive for RVF from 2004–2015 under each control scenario on Grande Comore (red), Mohéli (blue), Anjouan (green) and Mayotte (purple) compared with the full transmission model. Distributions were generated from 1,000 realisations of each scenario.

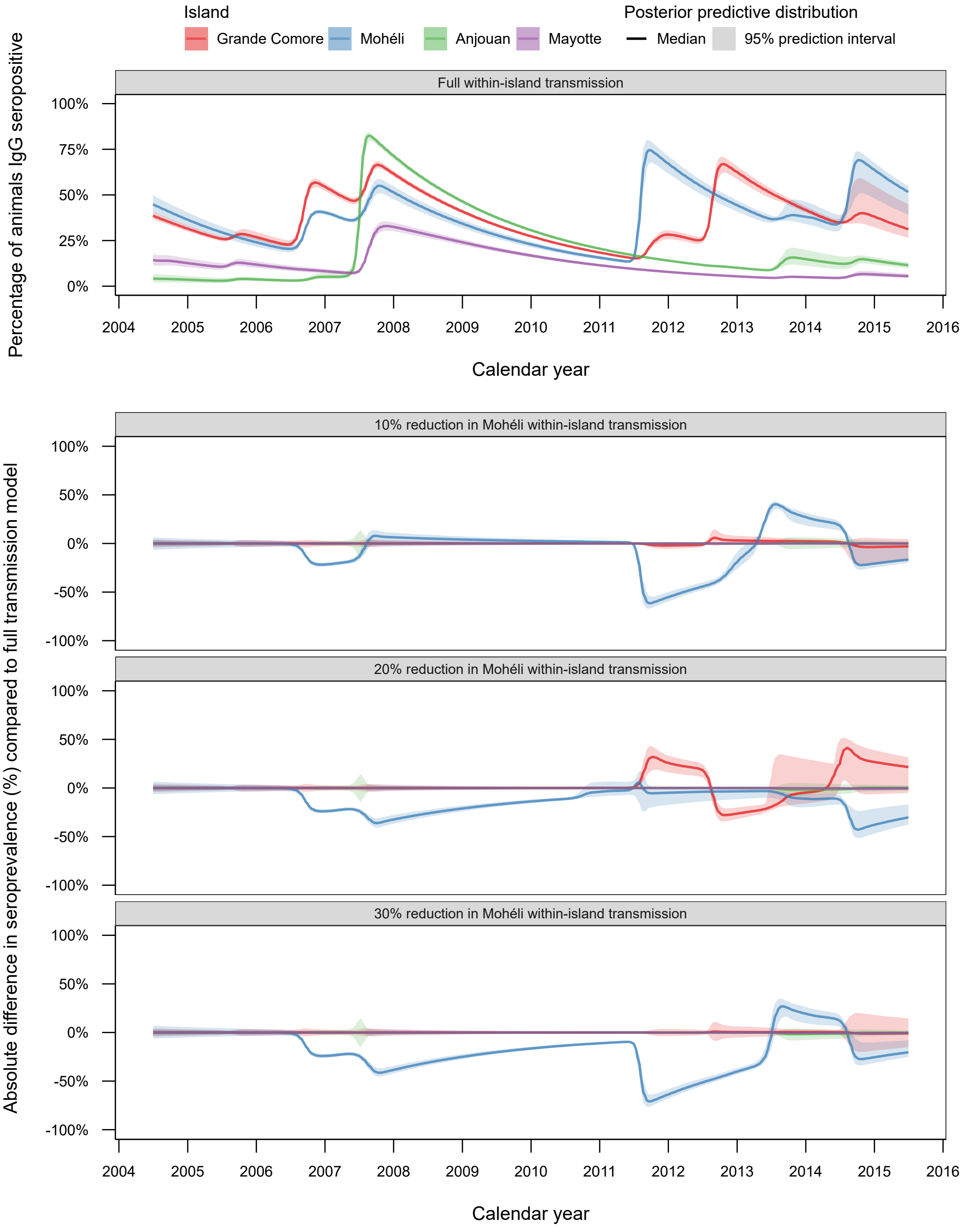

Supplementary Figure 10. **Effect of reducing within-island transmission of Mohéli on seroprevalence from 2004–2015.** The best fitted model (Model 3b) was simulated with different reductions in the transmission rate on Mohéli: 10%, 20% and 30%. Shown is the absolute difference in the percentage of animals IgG seropositive for RVF from 2004–2015 under each control scenario on Grande Comore (red), Mohéli (blue), Anjouan (green) and Mayotte (purple) compared with the full transmission model. Distributions were generated from 1,000 realisations of each scenario.

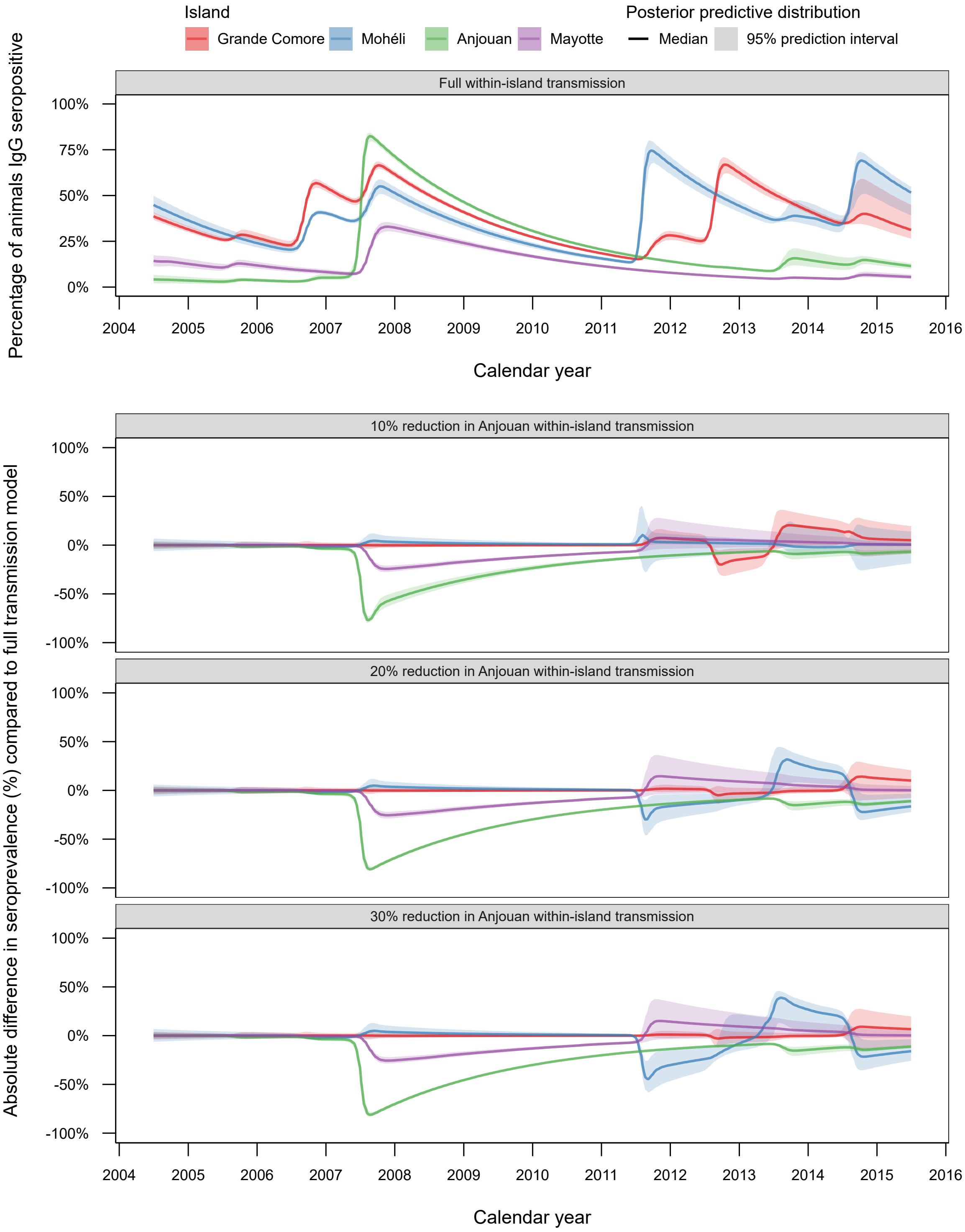

Supplementary Figure 11. **Effect of reducing within-island transmission of Anjouan on seroprevalence from 2004–2015.** The best fitted model (Model 3b) was simulated with different reductions in the transmission rate on Anjouan: 10%, 20% and 30%. Shown is the absolute difference in the percentage of animals IgG seropositive for RVF from 2004–2015 under each control scenario on Grande Comore (red), Mohéli (blue), Anjouan (green) and Mayotte (purple) compared with the full transmission model. Distributions were generated from 1,000 realisations of each scenario.

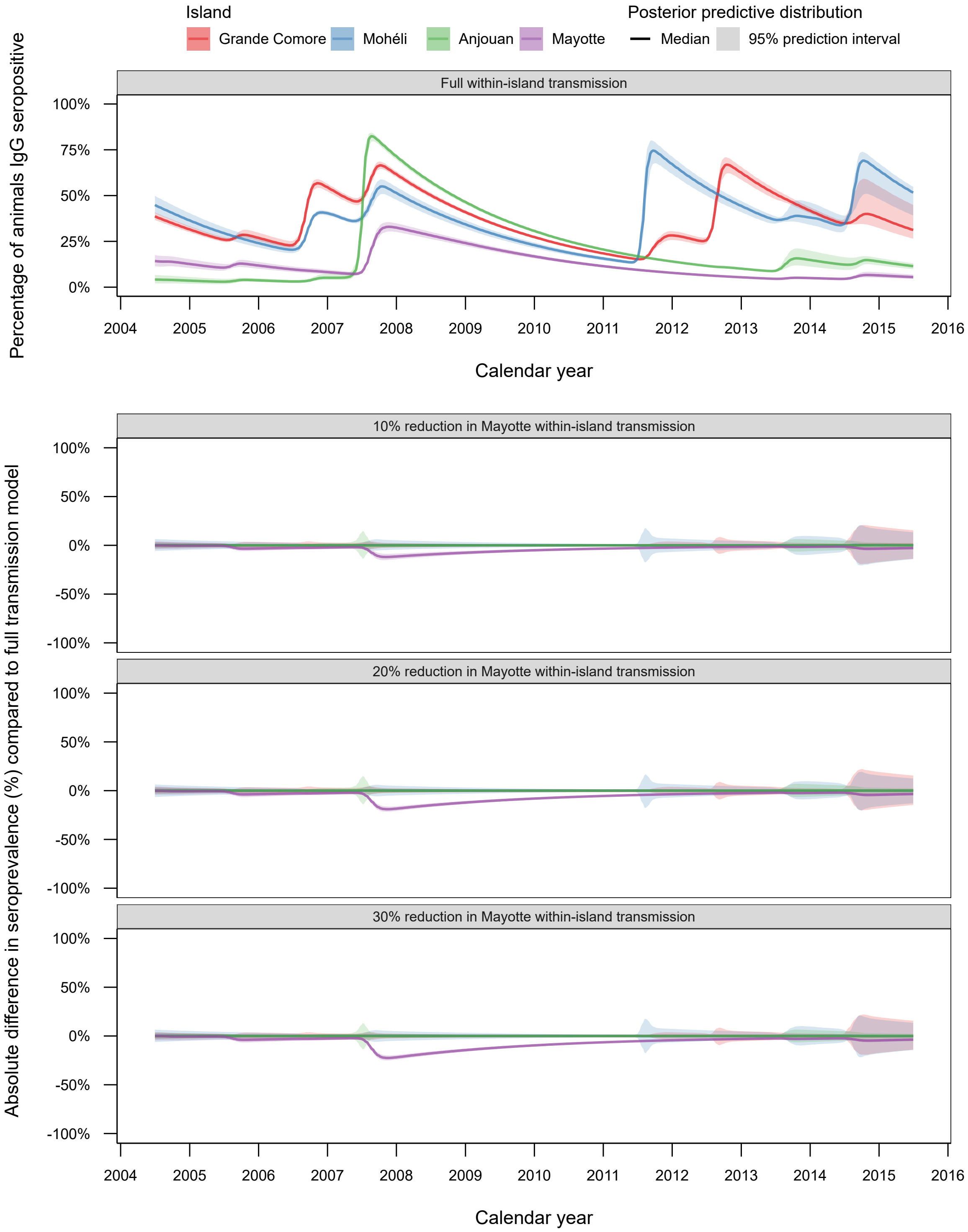

Supplementary Figure 12. **Effect of reducing within-island transmission of Mayotte on seroprevalence from 2004–2015.** The best fitted model (Model 3b) was simulated with different reductions in the transmission rate on Mayotte: 10%, 20% and 30%. Shown is the absolute difference in the percentage of animals IgG seropositive for RVF from 2004–2015 under each control scenario on Grande Comore (red), Mohéli (blue), Anjouan (green) and Mayotte (purple) compared with the full transmission model. Distributions were generated from 1,000 realisations of each scenario.

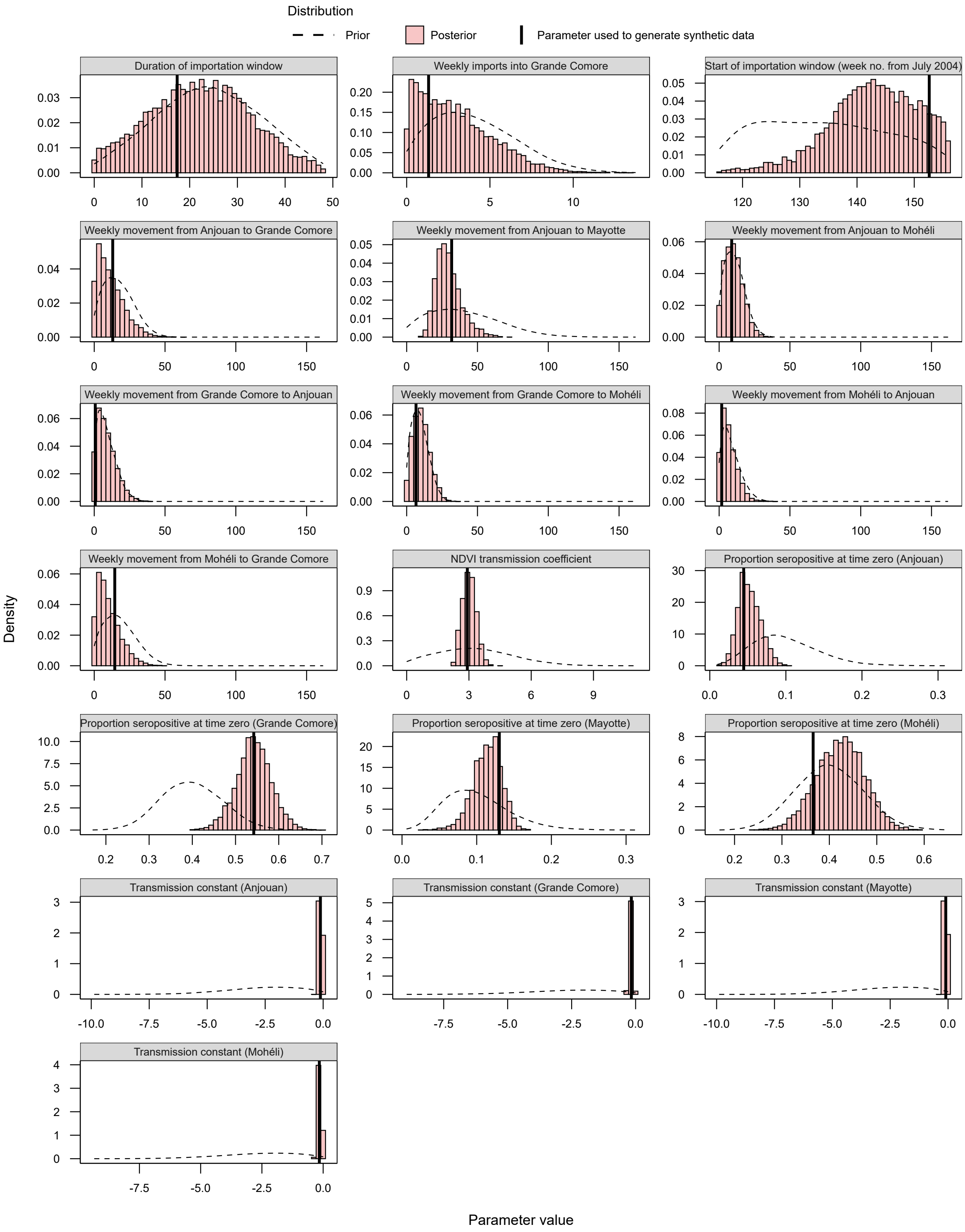

Supplementary Figure 13. **Posterior of estimated parameters with uninformative priors for weekly inter-island movements.** To investigate if serological data may inform movement parameters in multi-insular systems with little information on movement dynamics, the metapopulation model was fitted to synthetic data with the standard deviation of movement priors (Supplementary Table 4) multiplied by 10. Synthetic data was generated from the model using parameters values sampled from their respective prior. The model was fit back to this data using MCMC. Shown is the posterior distribution of each parameter of interest (bars) compared with the parameter value used to generate the synthetic data (vertical lines). Distributions shown were generated from 10,000 samples from the posterior of the fitted models.

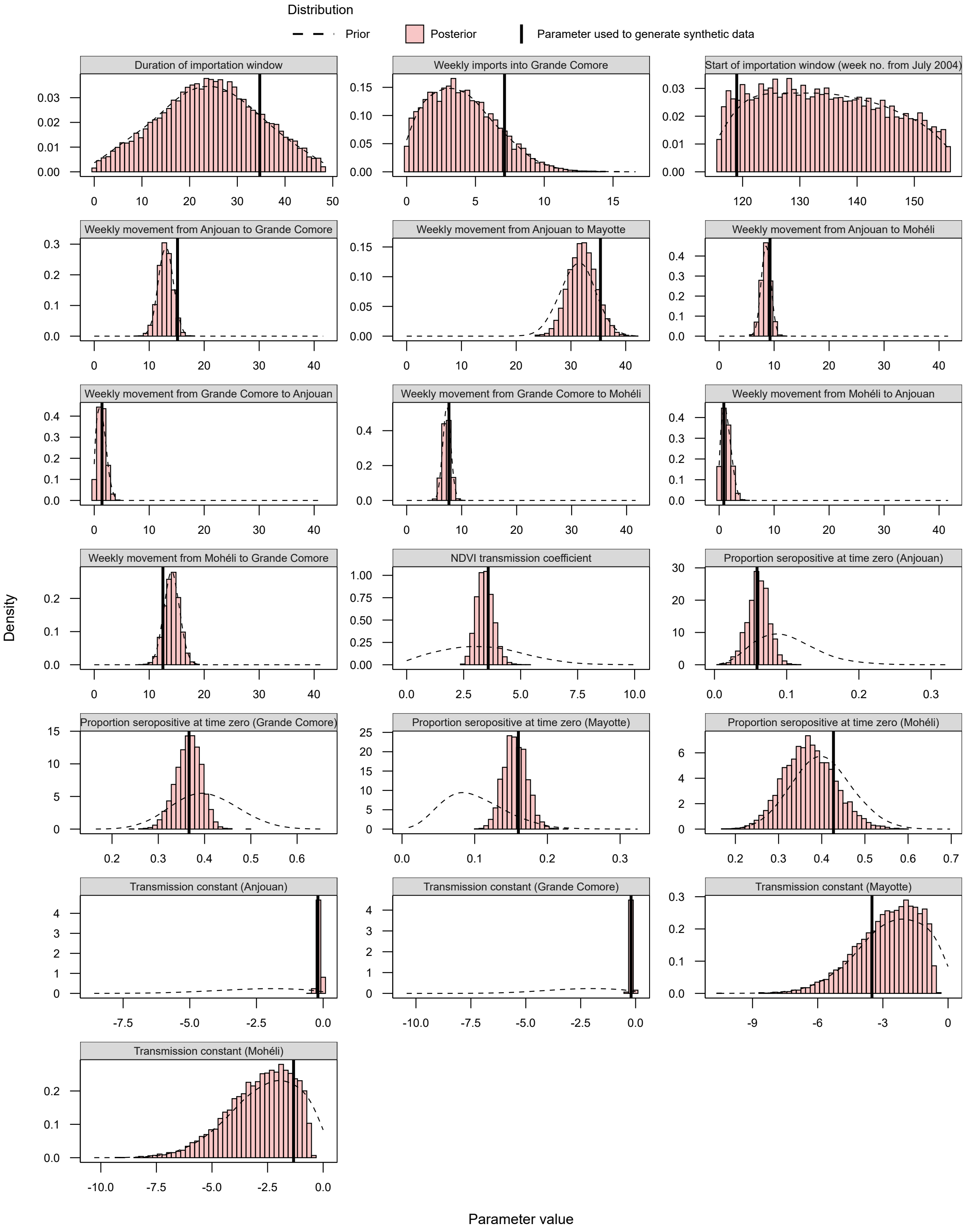

Supplementary Figure 14. **Posterior of estimated parameters in fitting to synthetic data #01.** Synthetic data was generated from the model using parameters values sampled from their respective prior. The model was fit back to the data using MCMC. Shown is the posterior distribution of each parameter of interest (bars) compared with the parameter value used to generate the synthetic data (vertical lines). In synthetic data #01, there were 437,239 livestock infections. Distributions shown were generated from 10,000 samples from the posterior of the fitted models.

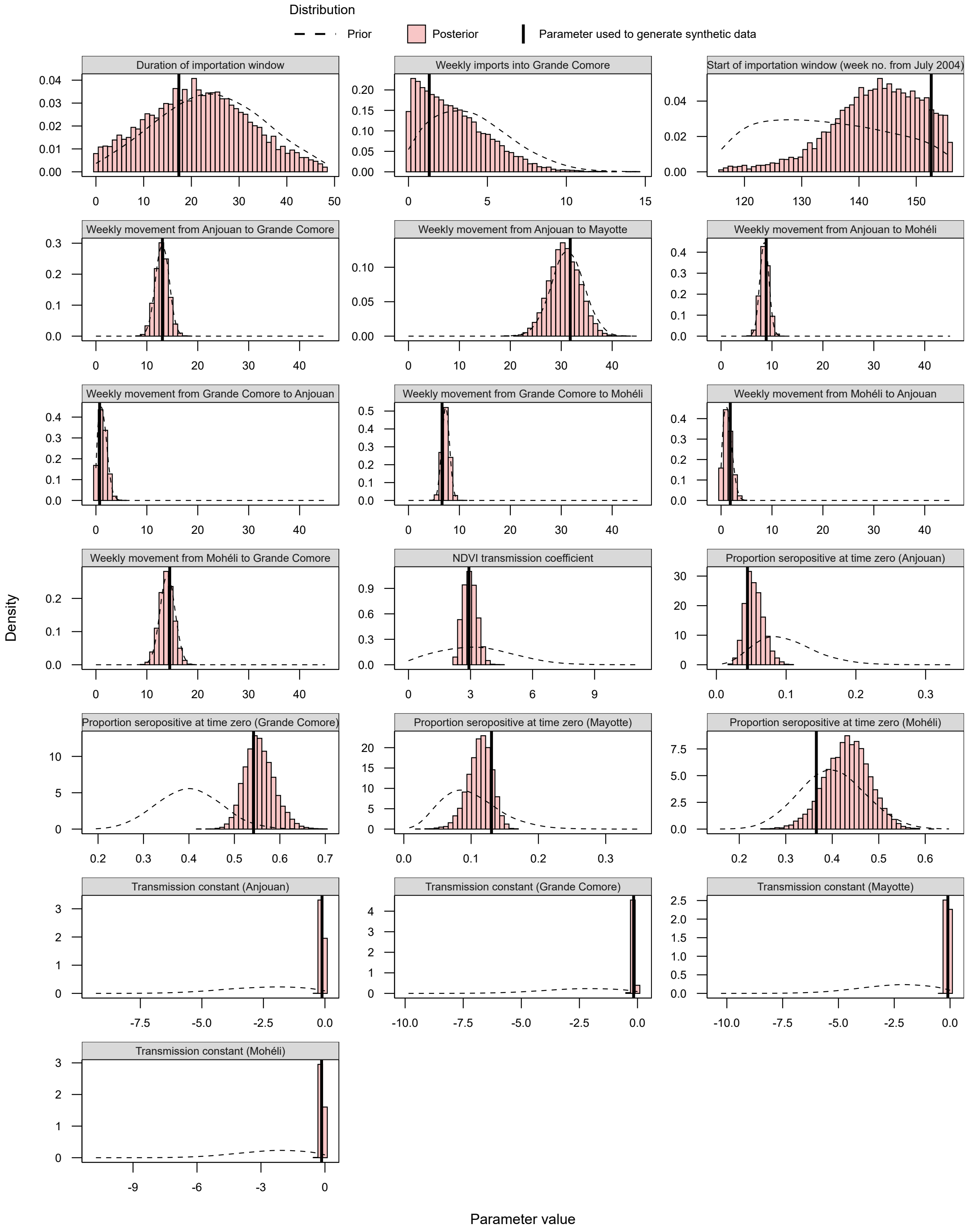

Supplementary Figure 15. **Posterior of estimated parameters in fitting to synthetic data #02.** Synthetic data was generated from the model using parameters values sampled from their respective prior. The model was fit back to the data using MCMC. Shown is the posterior distribution of each parameter of interest (bars) compared with the parameter value used to generate the synthetic data (vertical lines). In synthetic data #02, there were 384,948 livestock infections. Distributions shown were generated from 10,000 samples from the posterior of the fitted models.

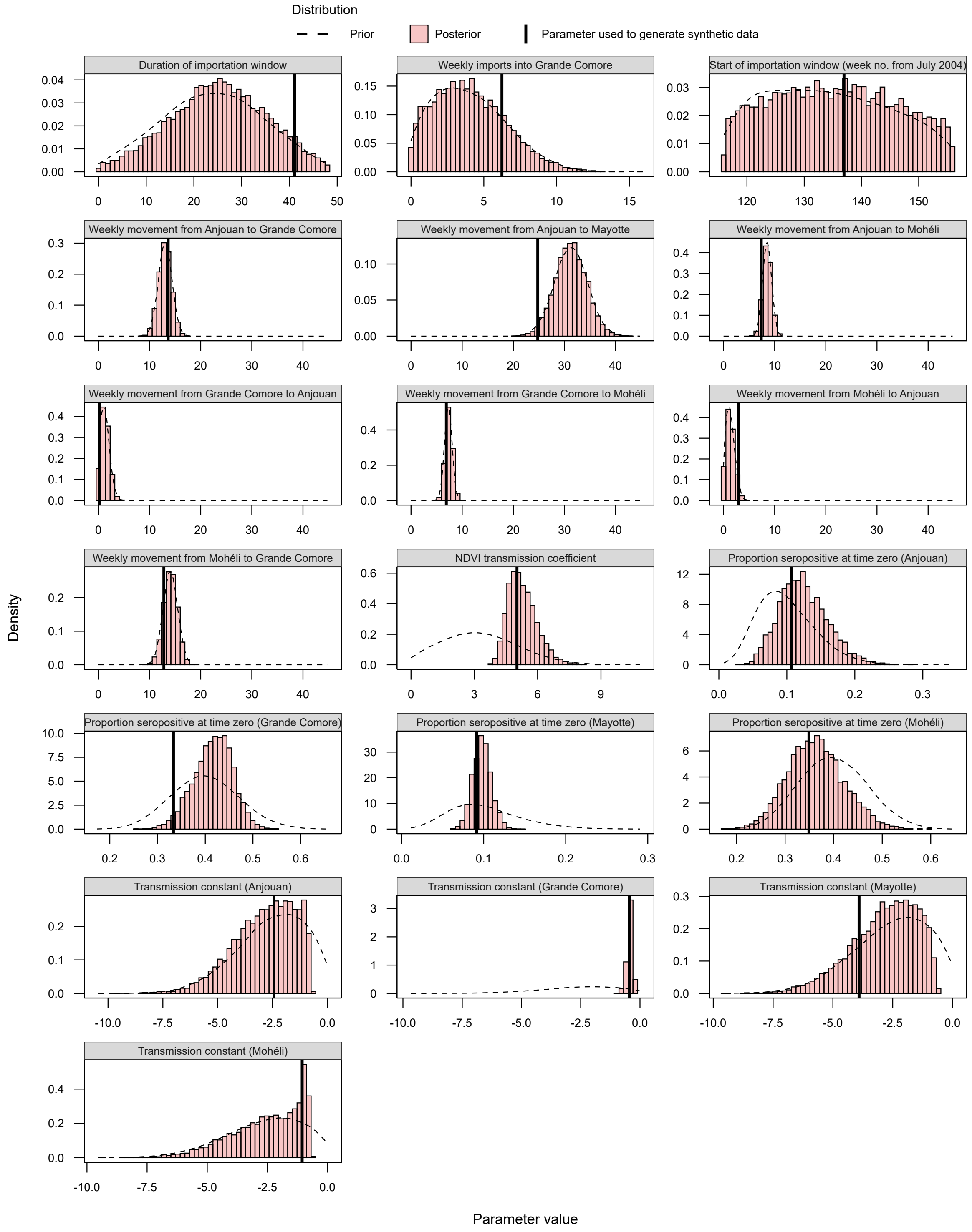

Supplementary Figure 16. **Posterior of estimated parameters in fitting to synthetic data #03.** Synthetic data was generated from the model using parameters values sampled from their respective prior. The model was fit back to the data using MCMC. Shown is the posterior distribution of each parameter of interest (bars) compared with the parameter value used to generate the synthetic data (vertical lines). In synthetic data #03, there were 306,277 livestock infections. Distributions shown were generated from 10,000 samples from the posterior of the fitted models.

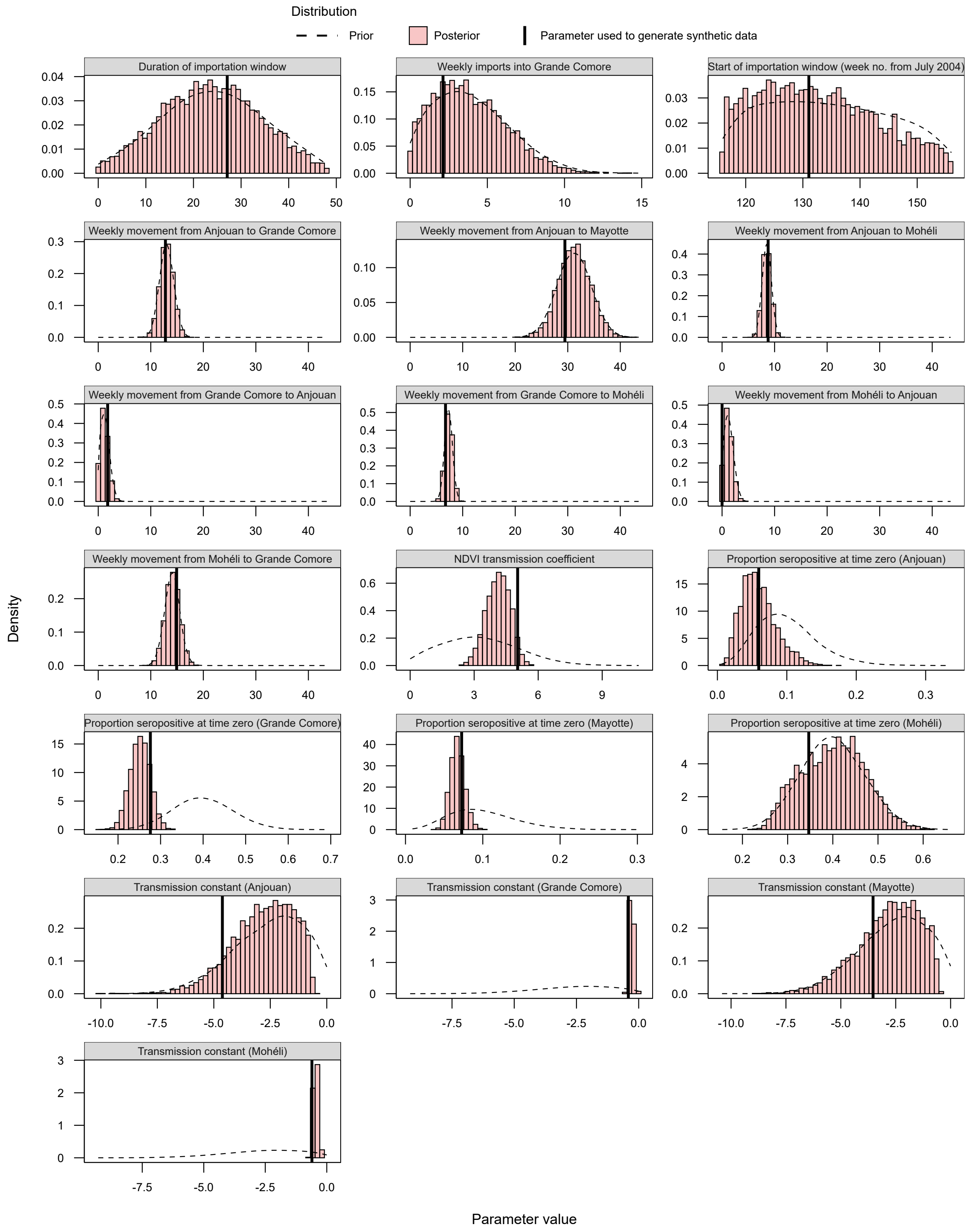

Supplementary Figure 17. **Posterior of estimated parameters in fitting to synthetic data #04.** Synthetic data was generated from the model using parameters values sampled from their respective prior. The model was fit back to the data using MCMC. Shown is the posterior distribution of each parameter of interest (bars) compared with the parameter value used to generate the synthetic data (vertical lines). In synthetic data #04, there were 326,461 livestock infections. Distributions shown were generated from 10,000 samples from the posterior of the fitted models.

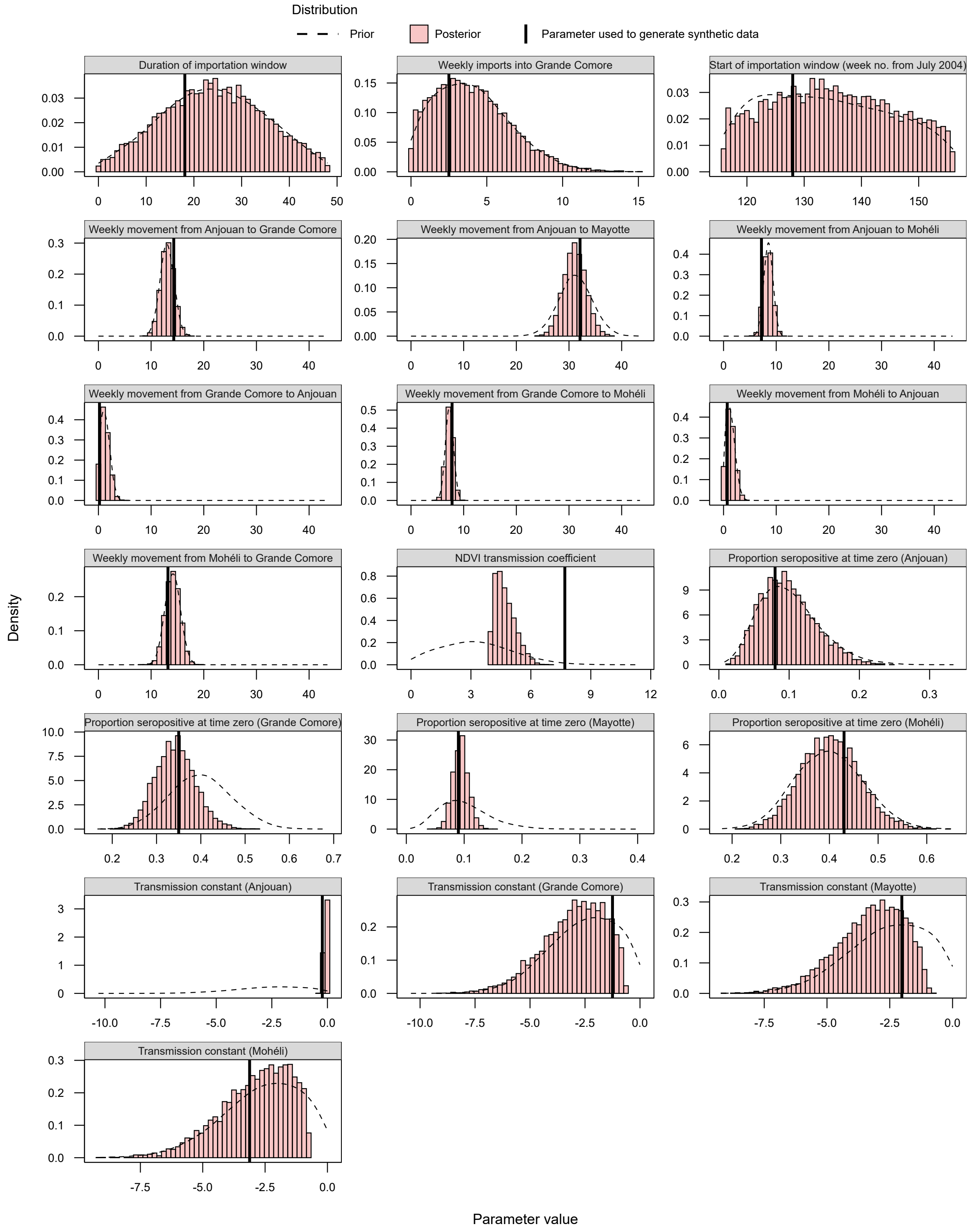

Supplementary Figure 18. **Posterior of estimated parameters in fitting to synthetic data #05.** Synthetic data was generated from the model using parameters values sampled from their respective prior. The model was fit back to the data using MCMC. Shown is the posterior distribution of each parameter of interest (bars) compared with the parameter value used to generate the synthetic data (vertical lines). In synthetic data #05, there were 123,208 livestock infections. Distributions shown were generated from 10,000 samples from the posterior of the fitted models.

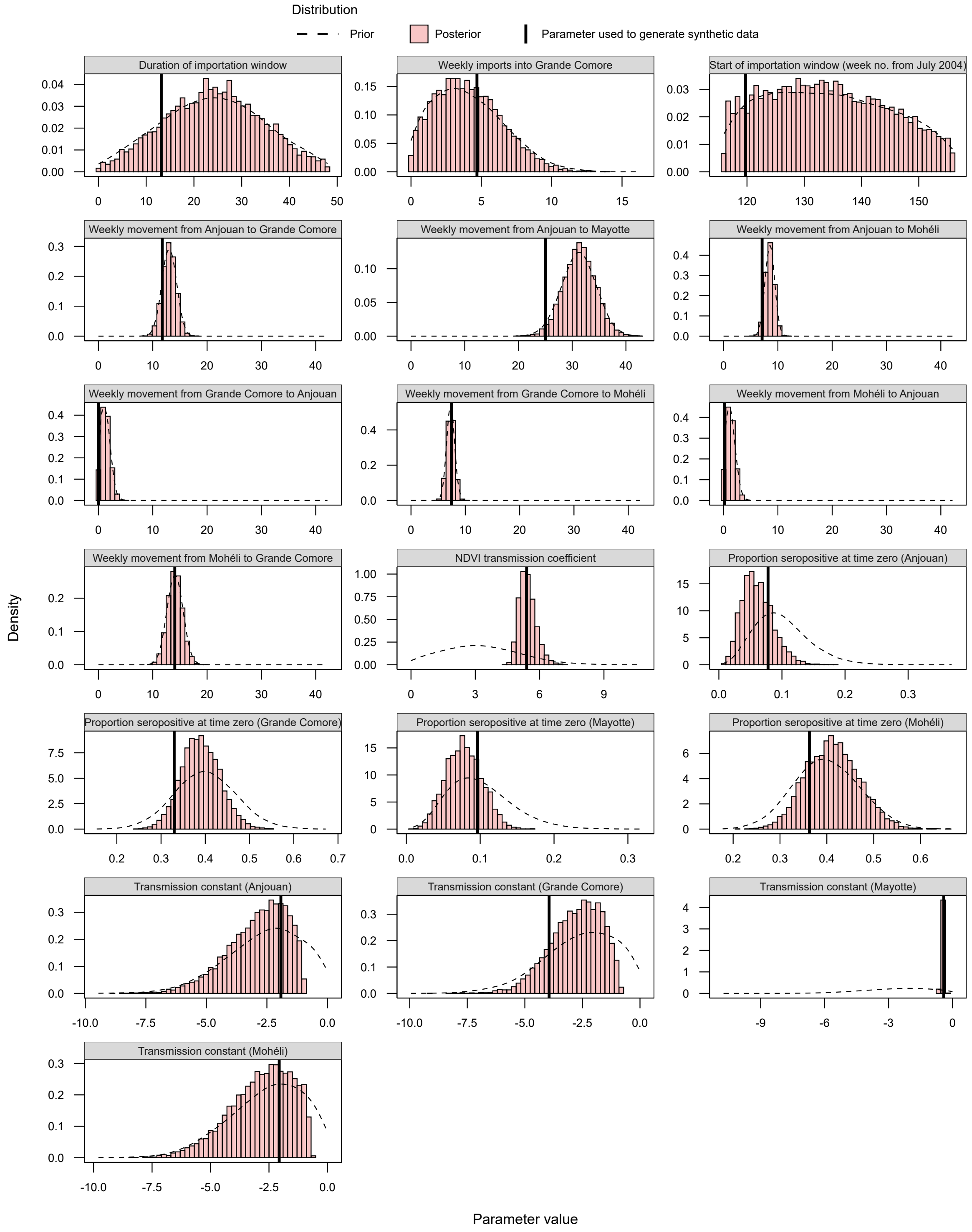

Supplementary Figure 19. **Posterior of estimated parameters in fitting to synthetic data #06.** Synthetic data was generated from the model using parameters values sampled from their respective prior. The model was fit back to the data using MCMC. Shown is the posterior distribution of each parameter of interest (bars) compared with the parameter value used to generate the synthetic data (vertical lines). In synthetic data #06, there were 30,369 livestock infections. Distributions shown were generated from 10,000 samples from the posterior of the fitted models.

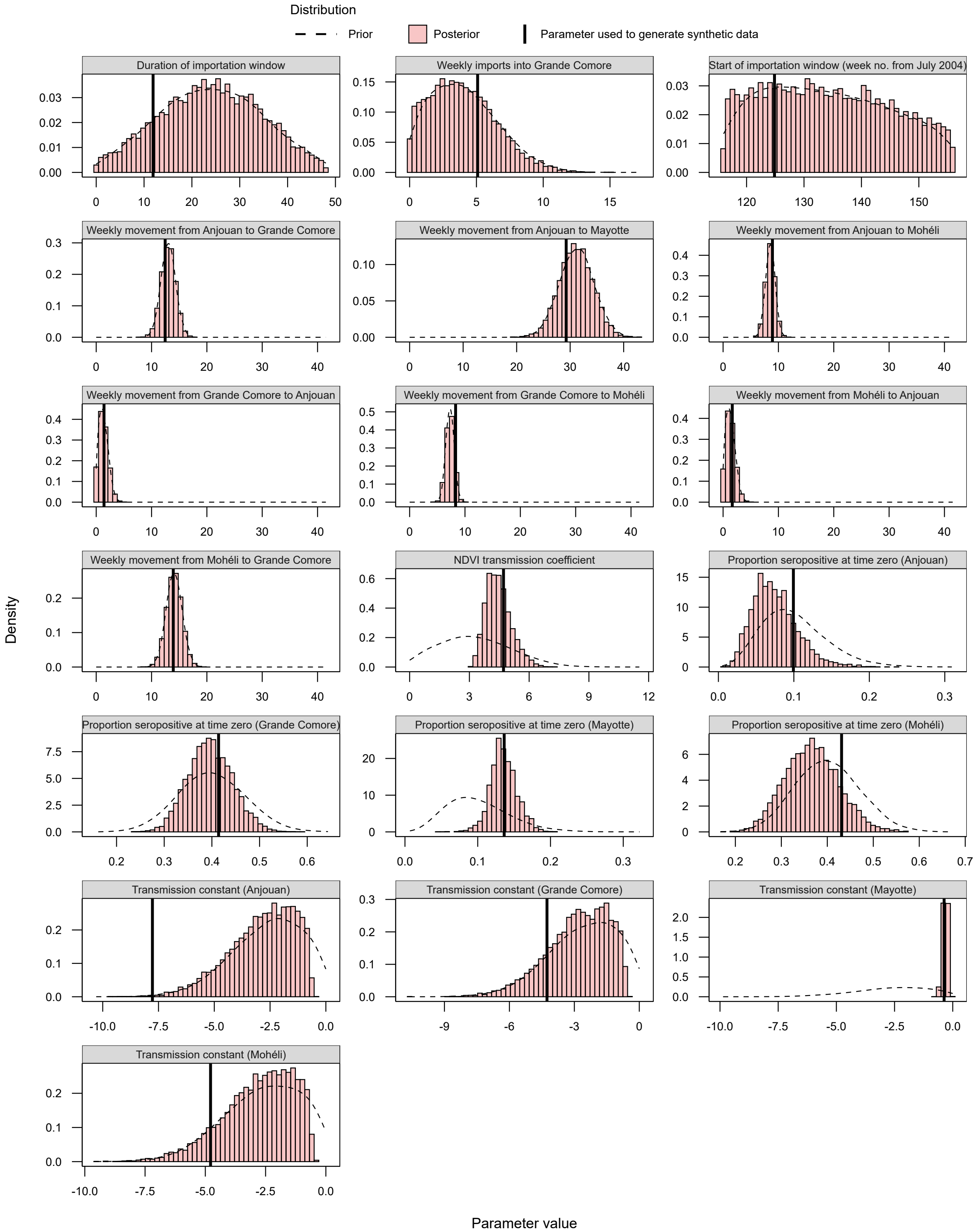

Supplementary Figure 20. **Posterior of estimated parameters in fitting to synthetic data #07.** Synthetic data was generated from the model using parameters values sampled from their respective prior. The model was fit back to the data using MCMC. Shown is the posterior distribution of each parameter of interest (bars) compared with the parameter value used to generate the synthetic data (vertical lines). In synthetic data #07, there were 25,272 livestock infections. Distributions shown were generated from 10,000 samples from the posterior of the fitted models.

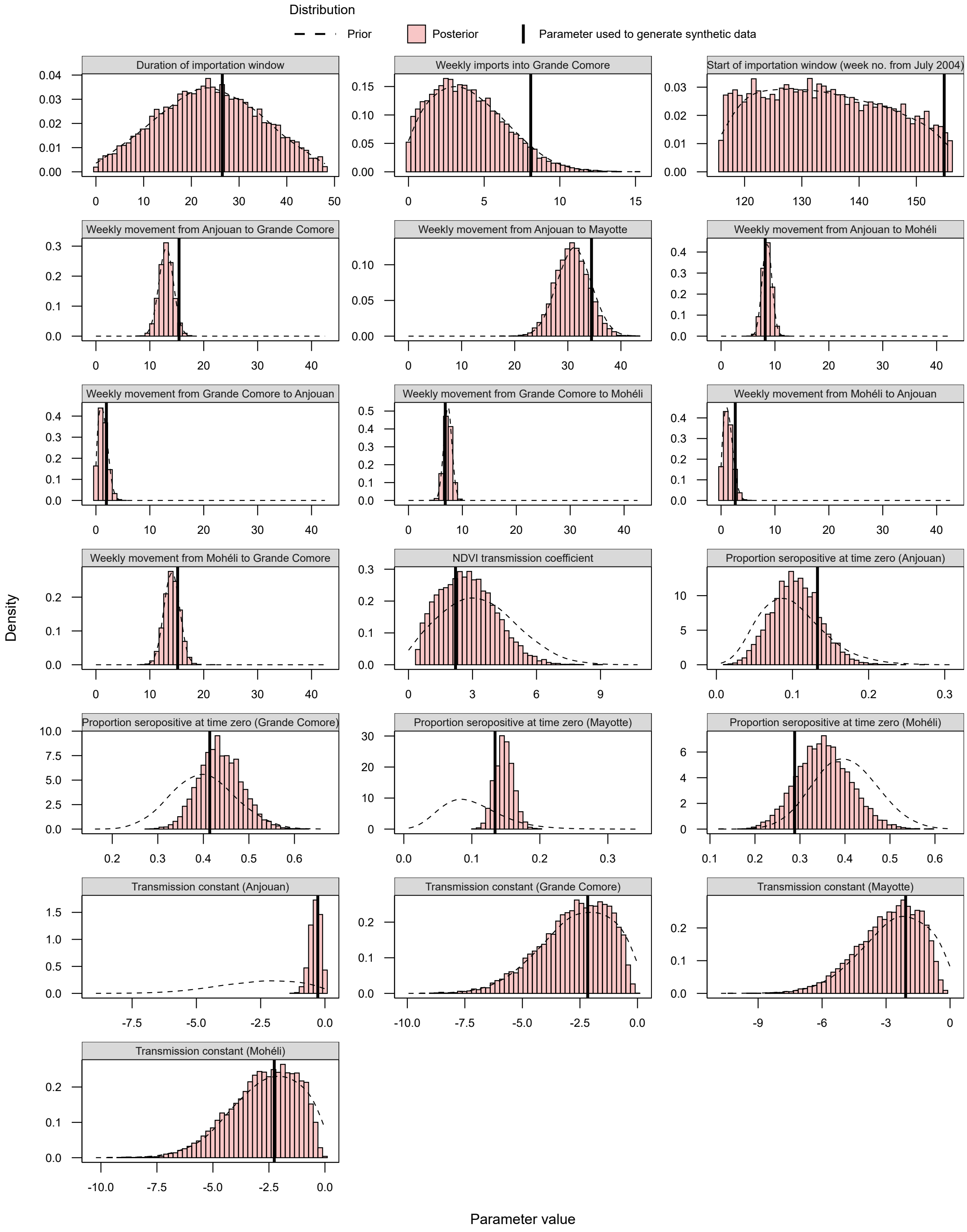

Supplementary Figure 21. **Posterior of estimated parameters in fitting to synthetic data #08.** Synthetic data was generated from the model using parameters values sampled from their respective prior. The model was fit back to the data using MCMC. Shown is the posterior distribution of each parameter of interest (bars) compared with the parameter value used to generate the synthetic data (vertical lines). In synthetic data #08, there were 13,286 livestock infections. Distributions shown were generated from 10,000 samples from the posterior of the fitted models.

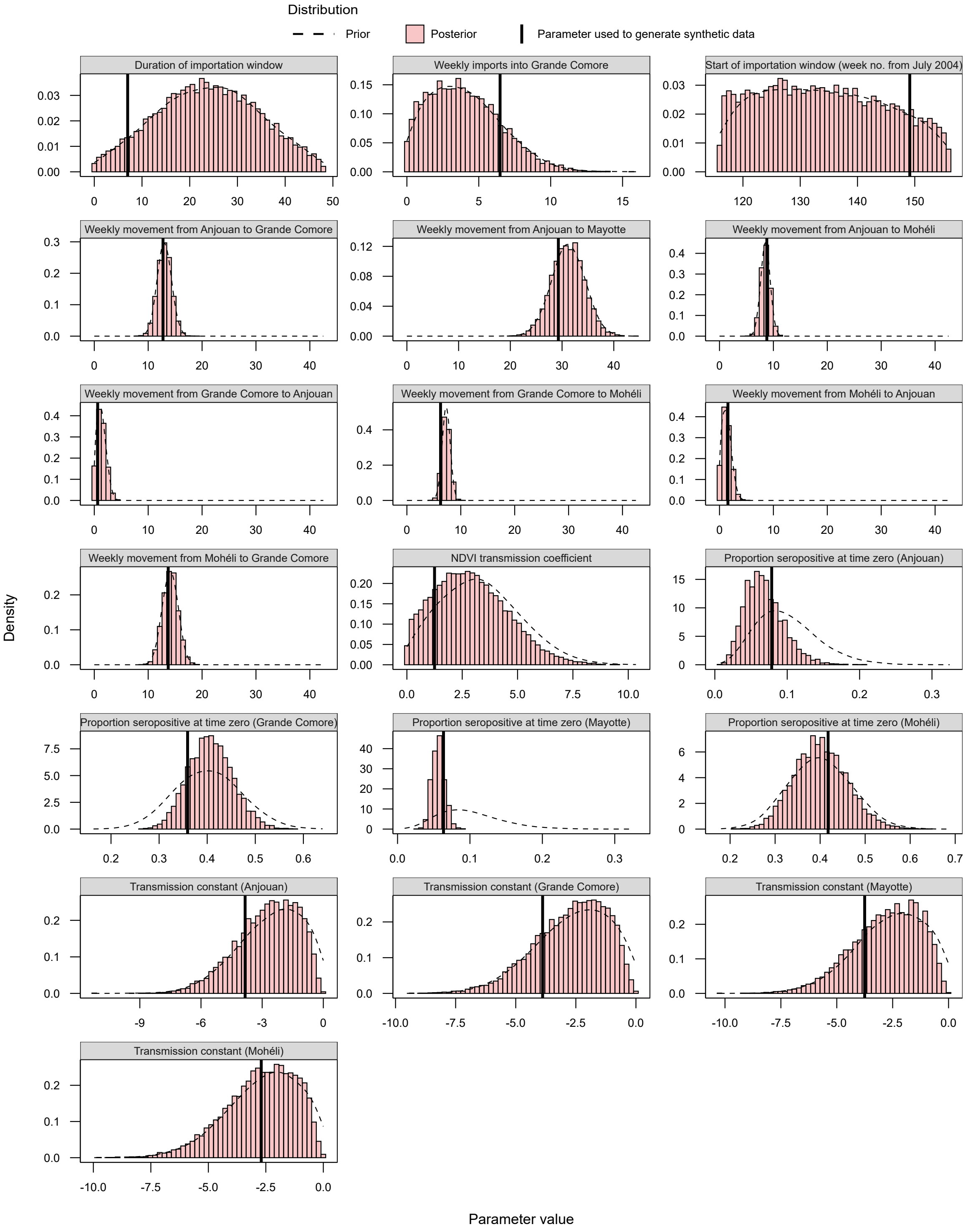

Supplementary Figure 22. **Posterior of estimated parameters in fitting to synthetic data #09.** Synthetic data was generated from the model using parameters values sampled from their respective prior. The model was fit back to the data using MCMC. Shown is the posterior distribution of each parameter of interest (bars) compared with the parameter value used to generate the synthetic data (vertical lines). In synthetic data #09, there were 228 livestock infections. Distributions shown were generated from 10,000 samples from the posterior of the fitted models.

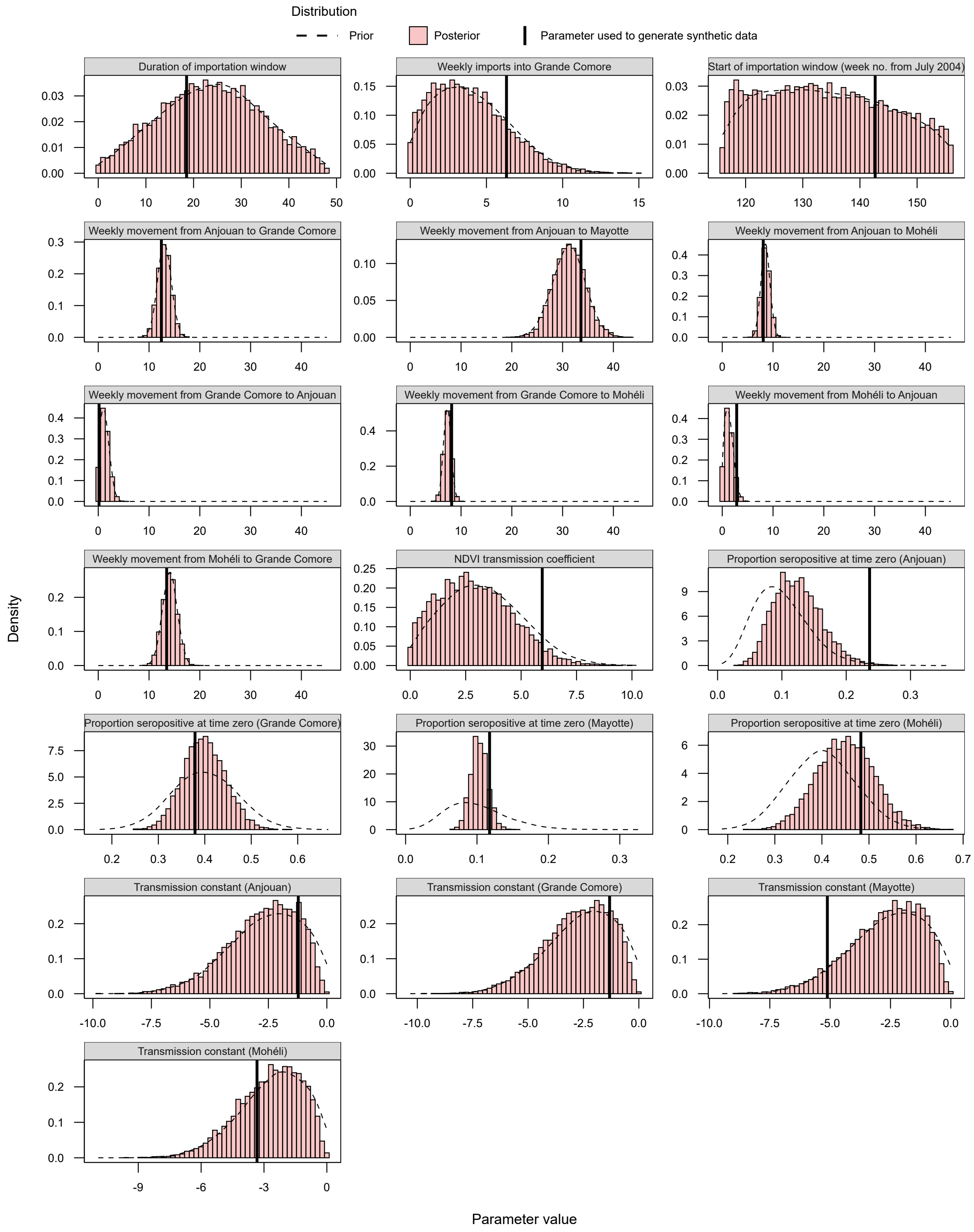

Supplementary Figure 23. **Posterior of estimated parameters in fitting to synthetic data #10.** Synthetic data was generated from the model using parameters values sampled from their respective prior. The model was fit back to the data using MCMC. Shown is the posterior distribution of each parameter of interest (bars) compared with the parameter value used to generate the synthetic data (vertical lines). In synthetic data #10, there were 211 livestock infections. Distributions shown were generated from 10,000 samples from the posterior of the fitted models.
