# Supplementary material for: Modelling the persistence and control of Rift Valley fever virus in a spatially heterogeneous landscape
Source: Nat Commun. 2021 Sep 22;12:5593. doi: 10.1038/s41467-021-25833-8 (PMC8458460; doi:10.1038/s41467-021-25833-8)
Supplement: Supplementary file 3 — Reporting Summary [file 41467_2021_25833_MOESM3_ESM.pdf]

nature research

Corresponding author(s): Warren Tennant

Last updated by author(s): Aug 20, 2021

Reporting Summary

Nature Research wishes to improve the reproducibility of the work that we publish. This form provides structure for consistency and transparency in reporting. For further information on Nature Research policies, see our Editorial Policies and the Editorial Policy Checklist.

Statistics

For all statistical analyses, confirm that the following items are present in the figure legend, table legend, main text, or Methods section.

n/a	Confirmed	
☐	☒	The exact sample size (n) for each experimental group/condition, given as a discrete number and unit of measurement
☐	☒	A statement on whether measurements were taken from distinct samples or whether the same sample was measured repeatedly
☒	☐	The statistical test(s) used AND whether they are one- or two-sided<br>*Only common tests should be described solely by name; describe more complex techniques in the Methods section.*
☐	☒	A description of all covariates tested
☐	☒	A description of any assumptions or corrections, such as tests of normality and adjustment for multiple comparisons
☐	☒	A full description of the statistical parameters including central tendency (e.g. means) or other basic estimates (e.g. regression coefficient) AND variation (e.g. standard deviation) or associated estimates of uncertainty (e.g. confidence intervals)
☒	☐	For null hypothesis testing, the test statistic (e.g. F, t, r) with confidence intervals, effect sizes, degrees of freedom and P value noted<br>*Give P values as exact values whenever suitable.*
☐	☒	For Bayesian analysis, information on the choice of priors and Markov chain Monte Carlo settings
☐	☒	For hierarchical and complex designs, identification of the appropriate level for tests and full reporting of outcomes
☒	☐	Estimates of effect sizes (e.g. Cohen's d, Pearson's r), indicating how they were calculated

Our web collection on statistics for biologists contains articles on many of the points above.

Software and code

Policy information about availability of computer code

Data collection	No software was used for data collection; all data in our study was secondary, which was collected on paper documents.
Data analysis	Metapopulation model simulation, model fitting and parameter estimation (the adaptive Markov Chain Monte Carlo Metropolis-Hastings random walk algorithm) and forecasting and control scenarios were all performed using custom-written code in C++14 using the GNU Scientific Library (GSL) version 2.6. Results were analysed and figures produced using R version 3.6.3 (2020-02-29) and tidyverse (version 1.3.0) library. All code is freely available at github.com/wtennant/rvf_comoros

For manuscripts utilizing custom algorithms or software that are central to the research but not yet described in published literature, software must be made available to editors and reviewers. We strongly encourage code deposition in a community repository (e.g. GitHub). See the Nature Research guidelines for submitting code & software for further information.

Data

Policy information about availability of data

All manuscripts must include a data availability statement. This statement should provide the following information, where applicable:

- Accession codes, unique identifiers, or web links for publicly available datasets
- A list of figures that have associated raw data
- A description of any restrictions on data availability

Summarised data used in our study, alongside a full description of the data, is available at github.com/wtennant/rvf_comoros. These data are openly accessible, and are presented in Figure 2 and Supplementary Figures 1–4.

Field-specific reporting

Please select the one below that is the best fit for your research. If you are not sure, read the appropriate sections before making your selection.

☐ Life sciences ☐ Behavioural & social sciences ☒ Ecological, evolutionary & environmental sciences

For a reference copy of the document with all sections, see nature.com/documents/nr-reporting-summary-flat.pdf

Ecological, evolutionary & environmental sciences study design

All studies must disclose on these points even when the disclosure is negative.

Study description	Our study was quantitative and sought to estimate the importance of environmental variables and livestock movement in RVF persistence and spatial spread in the Comoros archipelago – a network of islands, repeatedly affected by RVF, located in the south-west Indian Ocean. This included a mathematical model that (i) accounts for climate, animal movements and livestock susceptibility, (ii) considers spatial heterogeneity of these factors, and (iii) is fitted in a Bayesian framework to previously published RVF serological data from the Comoros archipelago.<br><br>The experimental unit for the serological data was the animal (livestock head), located on four islands from the Indian Ocean (namely Grande Comore, Mohéli, Anjouan and Mayotte). This network of four islands was chosen because they have been repeatedly affected by RVF over several years and constituted an ideal setting to study viral persistence between them because animals are moving across this network. In this study, we used secondary disease data (i.e. not collected for the purpose of this research), that were of relevance to study our research hypotheses. From those four islands, we used a total of 8423 livestock (cattle, sheep and goats) sera tested for RVF seropositivity between 2004 and 2015 were collated. More specifically, 2191 samples were collected in Grande Comore, 475 in Mohéli, 857 in Anjouan and 4900 in Mayotte.
Research sample	The data used in the model were RVF IgG prevalence in livestock (goat, sheep, and cattle) sera from four islands, collected as part as epidemiological investigations (longitudinal and cross-sectional surveys). When available, the age of the animal (in years) was used, otherwise animals were categorised as either young or adult. In Grande Comore, Moheli and Anjouan, animals were randomly sampled from herds selected based on the willingness of the farmers to cooperate during the longitudinal study; animals were selected based on their age (cattle between 10 months and one year of age, and small ruminants between three to eight months of age). The number of samples per village was calculated based on a previously estimated prevalence with a relative precision of 20% and a confidence level of 95% giving a required minimum (Roger et al., 2011) In Mayotte, herds were randomly selected based on the local disease surveillance system for ruminants.<br><br>No primary data was collected for the purpose of our study. The serological data described above, which was used during model fitting, was previously published in:<br><br>Roger, M., et al. Rift Valley fever in ruminants, Republic of Comoros, 2009. Emerging infectious diseases, 17(7): 1319, 2011.<br>Roger, M., et al. Evidence for circulation of the Rift Valley fever virus among livestock in the union of Comoros. PLoS Negl Trop Dis, 8(7):e3045, 2014.<br>Metras, R., et al. The epidemiology of Rift Valley fever in Mayotte: insights and perspectives from 11 years of data. PLoS neglected tropical diseases, 10(6):e0004783, 2016.<br>Metras, R., et al. Drivers for Rift Valley fever emergence in Mayotte: a Bayesian modelling approach. PLoS neglected tropical diseases, 11(7):e0005767, 2017.
Sampling strategy	We collated all the RVF seroprevalence data in livestock previously collected on the four islands during the study period and used them in the analyses to fit to our mathematical model. That is, no primary data was collected for the purpose of our study. This secondary serological data was previously published in:<br><br>Roger, M., et al. Rift Valley fever in ruminants, Republic of Comoros, 2009. Emerging infectious diseases, 17(7): 1319, 2011.<br>Roger, M., et al. Evidence for circulation of the Rift Valley fever virus among livestock in the union of Comoros. PLoS Negl Trop Dis, 8(7):e3045, 2014.<br>Metras, R., et al. The epidemiology of Rift Valley fever in Mayotte: insights and perspectives from 11 years of data. PLoS neglected tropical diseases, 10(6):e0004783, 2016.<br>Metras, R., et al. Drivers for Rift Valley fever emergence in Mayotte: a Bayesian modelling approach. PLoS neglected tropical diseases, 11(7):e0005767, 2017.<br><br>As part of our study, we demonstrated that these sample sizes were sufficient to identify the ecological drivers responsible for Rift Valley fever virus transmission in the Comoros archipelago. To do this, we generated synthetic data of the same sample sizes using our metapopulation model and randomly sampled values for the parameters we sought to estimate (e.g. the effect of climate on transmission rates on each island). We then fitted our metapopulation model back to this synthetic data, finding that these parameter estimates were recoverable given the sample size of the data and model design.
Data collection	This secondary data was collected as part of national livestock disease surveillance programs by Union of Comoros and Mayotte Veterinary Services. More specifically, the livestock sera were collected via blood sampling by Veterinary Services, and animal health workers, as part as disease surveillance programmes and epidemiological studies. The laboratory work (i.e. sample centrifugation, ELISA assays) was conducted by laboratory workers from the Mayotte departmental laboratory and GDS (Mayotte), the veterinary services (Union of the Comoros) (sample centrifugation) or by CIRAD scientists (ELISA assays) on previous publications of the data:<br><br>Roger, M., et al. Rift Valley fever in ruminants, Republic of Comoros, 2009. Emerging infectious diseases, 17(7): 1319, 2011.

Roger, M., et al. Evidence for circulation of the Rift Valley fever virus among livestock in the union of Comoros. PLoS Negl Trop Dis, 8(7):e3045, 2014.
Metras, R., et al. The epidemiology of Rift Valley fever in Mayotte: insights and perspectives from 11 years of data. PLoS neglected tropical diseases, 10(6):e0004783, 2016.
Metras, R., et al. Drivers for Rift Valley fever emergence in Mayotte: a Bayesian modelling approach. PLoS neglected tropical diseases, 11(7):e0005767, 2017.

All persons involved in data collection and analysis were trained either by Veterinary Services (blood sample collection and centrifugation) or by CIRAD scientists (ELISA assays).

Timing and spatial scale

Cross-sectional surveys were conducted in the Union of Comoros in 2009, 2012, 20113 and 2105 by the aforementioned surveillance programs. In Mayotte, livestock prevalence was continuously monitored throughout the period from 2004 until 2016 as the result of the 'Systeme d'epidemiosurveillance animale a Mayotte' (SESAM) surveillance system. More specifically, the studies were conducted in Grande Comore during May to June 2009, April to June 2010, August 2010, November 2010, February 2011, May 2011, July 2011, August 2011, April to June 2013 and May 2015; in Mohéli during July 2009, April 2010, August 2010, November 2010, February 2011, May 2011, August 2011, May 2013 and May 2015; in Anjouan in June 2009, April to May 2010, August 2010, November 2010, February 2011, May 2011, August 2011, April 2013, July 2013 and May to June 2015; and in Mayotte throughout July 2004 to June 2015.

Data exclusions

No data was excluded as part of our study.

Reproducibility

To ensure reproducibility of our parameter estimates, each model in our study was fitted using eight independent chains of parameters each initialised at random locations in parameter space. Convergence of the chains was assessed through visual inspection of trace plots and calculation of the Gelman-Rubin statistic. For each of the five models in our study, these checks were successful and thus confirmed that our parameter estimates were reproducible.

We also fitted our model to synthetic data generated from the model itself with randomly chosen parameters. By fitting our metapopulation model back to the synthetic data, we showed that parameter estimates could be reliably recovered. All attempts at reliably recovering model parameters using this approach were successful. This gave confidence that our model fitting procedure could estimate these parameters when fitting to the empirical data.

Randomization

During primary data collection, animals were sampled based on a randomization procedure where possible, otherwise, convenience sampling was used.

We accounted for this sampling process within our model by explicitly including an observation process on top of our infection model. Here, samples were grouped into the month and year of data collection, age of the animal and location (i.e. island) of the animals. This observation model allowed us to statistically evaluate the likelihood of observing a given number of RVF IgG positive animals in each sampling group following a randomized sampling strategy.

All simulations and model fitting exercises presented in our paper used randomization (i.e. a random seed) to ensure that our results were unbiased.

Blinding

Blinding was not relevant to our study as all data used was secondary, which involved serological testing of livestock, or simulated.

Did the study involve field work? ☐ Yes ☒ No

Reporting for specific materials, systems and methods

We require information from authors about some types of materials, experimental systems and methods used in many studies. Here, indicate whether each material, system or method listed is relevant to your study. If you are not sure if a list item applies to your research, read the appropriate section before selecting a response.

Materials & experimental systems

n/a	Involved in the study	
☒	☐	Antibodies
☒	☐	Eukaryotic cell lines
☒	☐	Palaeontology and archaeology
☒	☐	Animals and other organisms
☒	☐	Human research participants
☒	☐	Clinical data
☒	☐	Dual use research of concern

Methods

n/a	Involved in the study	
☒	☐	ChIP-seq
☒	☐	Flow cytometry
☒	☐	MRI-based neuroimaging
